# Placental macrophages present distinct polarization pattern and effector functions depending on clinical onset of preeclampsia

**DOI:** 10.3389/fimmu.2022.1095879

**Published:** 2023-01-12

**Authors:** Monika Horvat Mercnik, Carolin Schliefsteiner, Herbert Fluhr, Christian Wadsack

**Affiliations:** ^1^Department of Obstetrics and Gynaecology, Medical University of Graz, Graz, Austria; ^2^BioTechMed-Graz, Graz, Austria

**Keywords:** hofbauer cell, preeclampsia, polarization, early onset preeeclampsia, late onset preeclampsia, inflammation, macrophage, human placenta

## Abstract

Hofbauer cells (HBCs) are resident macrophages of the human placenta, regulating immune tolerance and tissue homeostasis. HBCs of a normal placenta (CTR) exhibit mainly an anti-inflammatory M2 phenotype. Under exaggerated chronic inflammation during pregnancy, as in preeclampsia (PE), a phenotypic switch towards M1 polarization has been proposed. PE, defined as maternally derived syndrome can be distinguished into two different entities: early-onset (EO) preeclampsia and late-onset (LO) preeclampsia. Although the clinical presenting characteristics overlap, both can be identified by biochemical markers, heritability, and different maternal and fetal outcomes. To date, no study has specifically investigated polarization and phenotype of EO- and LO-PE HBCs and looked at possible changes in HBC functionality. Primary HBCs were isolated from CTR and PE placentae. First, *in vitro* morphological differences were observed between CTR and PE HBCs, with both PE groups exhibiting features of M1 macrophages alongside M2 forms. Interestingly, a different polarization pattern was observed between EO- and LO-PE HBCs. EO-PE HBCs develop a tissue remodeling M2 phenotype that is strongly shifted toward M1 polarization and showed a significant upregulation of CD86, TLR4, and HLA-DR. Furthermore, this pro-inflammatory signature is corroborated by higher expression of IRF5 and of *NOS2* (p ≤ 0.05). However, their M2 characteristics is reflected by significant TGF-β secretion and *ARG1* expression. In contrast, LO-PE HBCs developed a phagocytic CD209-low M2 phenotype in which the M1 pattern was not as pronounced as they downregulated the *NOS2* gene, but expressed increased levels of pro-inflammatory CD80 and TLR1 (p ≤ 0.05). The enhanced phagocytosis and MMP-9 secretion alongside the increased secretion of anti-inflammatory IL -4, IL -13 and TGF-β in both EO- and LO-PE HBCs suggests their adaptive role and plasticity in resolving inflammation and tissue homeostasis.

## 1 Introduction

Preeclampsia is a maternally derived inflammatory syndrome, affecting 4-5% of pregnancies worldwide. It is a leading cause of preterm delivery and intrauterine growth restriction, mainly due to the insufficient nutrient supply across the placenta and chronic hypoxia exposure of the fetus (1, 2). PE is clinically defined as *de novo* onset of hypertension (≥140/90 mmHg) accompanied by one or more of the following new-onset conditions: proteinuria, thrombocytopenia, renal failure, impaired liver function, pulmonary edema, neurological complications, or uteroplacental dysfunction, occurring after 20 weeks of gestation (1–3). Depending on the time of diagnosis, this syndrome can be divided into two subgroups, namely before (early-onset, EO) or after (late-onset, LO) 34 weeks of gestation (4–6). PE is a complex and heterogeneous disorder whose pathophysiological mechanisms are still not fully understood (7). Of note, different aetiology of EO- and LO-PE has been suggested. Briefly, EO-PE is associated with placental dysfunction and is more likely to affect the fetus (8, 9), whereas LO-PE is mediated by maternal factors, therefore more favourable for successful fetal outcome (10). The placenta in PE is characterized by profound morphological and functional alterations (11), due to poor placentation and placental ischemia (12, 13). In addition, placental dysfunction has been associated with an imbalanced immune function, excessive inflammation accompanied with increased production of pro-inflammatory factors, and simultaneously a decrease in the number of regulatory immune cells and anti-inflammatory cytokines, all together contributing to the development and progress of PE (14–16).

Both the maternal and feto-placental immune system play a crucial role in the development of pregnancy (16). Hence, in contrast to normal pregnancy, where the immune systems contribute to the maintenance of feto-maternal tolerance and placental development, a pro-inflammatory environment leads to excessive activation of innate immune cells and consequently to placental dysfunction and/or poor maternal vascular adaptation (16–19). Macrophages represent a diverse group of innate immune cells, vital for the regulation of inflammation, tissue homeostasis, and defence (20). Due to their remarkable plasticity that allows them fast and direct response to the stimuli and to the adaptive capability of their micro-environmental milieu (21), they are important key players in the progression of pregnancy and could be involved in the development and progression of PE (16, 19). Macrophages are keen to develop a broad spectrum of phenotypes along the M1 and M2 axis, which allows to divide the cells into defined classical M1 and M2 polarized groups (22, 23) The balance between the different polarization states often plays an important role in the resolution or progression of inflammation (24, 25). Their phenotypic heterogeneity is also reflected in their effector functions. In general, M1 macrophages are thought to be pro-inflammatory, while M2 macrophages limit inflammation and promote tissue repair, angiogenesis and homeostasis (26, 27).

Hofbauer cells (HBCs) are placental macrophages residing from day 18 after conception (28) in the chorionic villi of the human placenta (29). In a normal pregnancy HBCs are M2 polarized (26, 30–33) long spindled cells with large vacuoles (34). Due to their phenotypic heterogeneity, HBCs fulfil a variety of functions (35). As placental immune cells, they exhibit micro-biocidal activity (36, 37) and promote maternal tolerance towards the fetus (38). They are known to engage in tight and specific interactions with surrounding placental cells, therefore promoting trophoblast function (39, 40), tissue remodeling (36) and angiogenesis (26, 36, 41, 42). Perturbations in the homeostatic functions of HBCs are often associated with inflammation (43–46) and infection (47). Despite their crucial role in placental tissue, knowledge about the role of HBCs in PE is still lacking. A deeper understanding of HBC function offers the potential for therapeutical immune manipulation during compromised pregnancies in relation to gestational age, which determines both maternal and perinatal outcomes.

This study aimed to investigate polarization and phenotypic differences of primary human HBCs isolated from normal and PE placentae. In addition, we tested whether changes of the HBC phenotype might be linked to altered functionality, specifically to phagocytosis, tissue remodeling and the ability of macrophages to activate feto-placental endothelial cells (fpEC). Further, as gestational age has been identified as the most important clinical variable, we hypothesized that stratification of PE (EO-PE vs LO-PE) may account for the observed functional changes of HBCs within each group. These findings, while somewhat preliminary (due to case numbers), demonstrate that the inflammatory placental environment of EO-PE alters the immunoregulatory phenotype of HBCs with an increased pro-inflammatory M1 signature. Interestingly, LO-PE HBCs remained M2 polarized cells, but with a different polarization pattern as controls.

## 2 Materials and methods

### 2.1 Study population

In this study preeclampsia was defined according to the guidelines of the American College of Obstetricians and Gynaecologists as already mentioned above (1, 2). The institutional ethics committee of the Medical University of Graz (29-319 ex 16/17) approved the study. Subjects included in the study signed an informed consent form before participation, the characteristics of which are shown in the **Table 1**. Included placentae from singleton pregnancies were used within 30 minutes of caesarean section or vaginal delivery. PE was defined as a sustained blood pressure of 140/90 mm Hg or greater (on two occasions at least 4 hours apart) occurring after 20 weeks of gestation in a woman with previously normal blood pressure, accompanied by one or more of the following new onset conditions: proteinuria, thrombocytopenia, renal insufficiency, impaired liver function, pulmonary edema, neurological complications or uteroplacental dysfunction. Onset of PE was defined as early (EO, delivered and detected before 34 weeks of gestation) or late (LO, delivered and detected after 34 weeks of gestation) (1). Placentae from normal pregnancies served as controls.

**Table 1:** Subject characteristics of women and their offspring included in the study.

	CTR (n=22)	EO-PE (n=8)	LO-PE (n=6)
Age	29.4 ± 3.5	34.4 ± 3.6 **	34.4 ± 4.0 †
BMI before pregnancy (kg/m2)	22.5 ± 2.9	21.0 ± 2.1	23.4 ± 3.2
Week of gestation	38.7 ± 1.5	34.2 ± 1.1 ****	37.7 ± 1.1
Mode of the delivery	SP 10/CS 12	CS 8	SP 3/CS 3
Fetal sex	8♀ 14♂	4♀, 4♂	2♀, 4♂
Umbilical cord blood Arterial, pH	7.30 ± 0.06	7.31 ± 0.03	7.25 ± 0.09
Umbilical cord blood Venous, pH	7.37 ± 0.06	7.36 ± 0.02	7.34 ± 0.05
Placental weight (g)	616.7 ± 123.0	425.7 ± 63.7	506.7 ± 82.8
Birth weight (g)	3345 ± 398	1996 ± 404.7 ****	2955 ± 288.9
Birth weight percentile	49.2 ± 21.2	24.0 ± 18.7 **	34.3 ± 11.3
Systolic blood pressure (mmHg)	115.1 ± 6.9	162 ± 12.1****	153.2 ± 20.9 ††††
Diastolic blood pressure (mmHg)	72.9 ± 10.2	94.3 ± 8***	103 ± 13.3 ††††
sFlt-1 [pg/mL]	/	14472 ± 5083 ‡	8257 ± 2881
PlGF [pg/mL]	/	66.2 ± 24.3	81.6 ± 13.02
sFlt-1/PlGF [pg/mL]	/	248.7 ± 118.1	105.5 ± 51.9
Platelets [109/L]	218.3 ± 63.1	221.9 ± 106.3	191.8 ± 60.8
Uric acid [mg/dL]	/	6.3 ± 1.4	5.9 ± 0.7
AST [U/L]	/	21.0 ± 6.3	21.2 ± 4.2
ALT [U/L]	/	17.4± 5.6	12.7 ± 4.8

BMI, body-mass index; SP, spontaneous delivery; CS, caesarean section; All data are shown as mean ± SD. Statistical significance was assessed by one-way ANOVA with Tukey’s *post-hoc* test. If normality testing failed, Kruskal-Wallis test with Dunn’s *post-hoc* test was used. When comparing two groups’ Students t-test was used. **p ≤ 0.01, ***p ≤ 0.001 and ****p ≤ 0.0001; whereas *CTR vs EO-PE. †p ≤ 0.05 and † † † †p ≤ 0.0001; whereas †CTR vs LO-PE. ‡ represents comparison between EO-PE vs LO-PE; whereas ‡ p ≤ 0.05.

### 2.2 Isolation of HBCs

Primary HBCs were isolated according to a modified protocol as described by Tang et al. (48). To avoid contamination with decidual macrophages, the decidual membrane was removed before isolating HBCs. The villous tissue was dissected, washed in 0.4% saline solution (Fresenius, Cat #C924228), and finely minced. Between 60 and 100 g of the minced tissue was stored overnight at 4°C in 1 x phosphate buffered saline (PBS, Medicado, Cat #09-9400-100). On the next day, the tissue was digested with trypsin (0.25%, Sigma Aldrich, Cat #T4549) and DNase I (0.08 mg/ml; Roche, Cat #10104159001), followed by digestion with collagenase A (1 mg/ml; Roche, Cat #10103586001) and DNase I (0.08 mg/ml, Roche, Cat #10104159001). The cell suspension containing the HBCs was applied to a Percoll gradient (20-40%, Sigma Aldrich, Cat #P4937) and centrifuged unrestrained at 1000 g for 30 min. At this point, the HBCs appearing as bands between 30 and 35% gradient layers were aspirated and purified by negative selection using Dynabeads (Invitrogen, Cat #11033) coated with antibodies against epithelial growth factor receptor (EGFR, Santa Cruz, Cat #sc-120) and CD10 (Sigma Aldrich, Cat #SAB4700440). After immunopurification, cells were seeded in macrophage medium (MaM, ScienCell, Cat #SC1921) containing 5% FBS (ScienCell, Cat #SC1921), PenStrep (ScienCell, Cat #SC1921) and macrophage growth supplements (ScienCell, Cat #SC1921) at a cell density of 1x10^6^ cells/ml. Cells were cultured at 21% oxygen and 37°C. Quality control of the isolated HBCs was performed on the fixed cells after 6 days by immunocytochemistry for CD163 (Thermo Fischer Scientific, Cat #MA1-82342), CD90 (Dianova, Cat # DIA100), CD80 (Abcam, Cat #ab86473), CD68 (Dako, Cat #GA613), CD86 (Abcam, Cat #ab270719), CD206 (Novus, Cat #H00004360), CD209 (R&D Systems, Cat #MAB1621), and isotype control (Dako, Cat #X0931) as previously described by Schliefsteiner et al. (31). Cell culture images were obtained using brightfield microscope with a SC50 Olympus camera and CellSens software.

### 2.3 Immunohistochemistry

Tissue sections were taken from four different areas of placenta (reaching from chorionic plate to the decidual side and a central region of the placental disk) and fixed overnight in 4% neutral buffered paraformaldehyde solution. After paraffin embedding, tissue sections with a thickness of 5μm were mounted on glass slides. The paraffin was then removed with xylene and rehydrated in an ethanol dilution series. Antigen retrieval was performed using a citrate buffer (Gatt, Cat #403139070) adjusted to pH 6.0. UltraVision LP detection system (Thermo Fischer Scientific, Cat #TL125HL) was used for histochemical immunostaining. Tissue was incubated with Hydrogen Peroxide Block (Thermo Fischer Scientific, Cat #TL125HL) for 15 minutes and washed in TBE buffer (Gatt, Cat #403211370), followed by a 5-minute incubation with Ultra V protein block (Thermo Fischer Scientific, Cat #TL125HL). The primary antibody isotype control (1:200, Dako, Cat #X0931) and CD163 (1:200, Thermo Fischer Scientific, Cat #MA1-82342) were diluted in antibody diluent (Agilent, Dako, Cat #S0809) and incubated overnight at 4°C in a humidified chamber. After washing step, primary antibody enhancer (Thermo Fischer Scientific, Cat #TL125HL) was applied for 20 minutes. After another washing step samples were incubated with Large HRP Polymer (Thermo Fischer Scientific, Cat #TL125HL) solution for 30 minutes, followed by intensive washing and incubation with AEC Chromogen Solution (Abcam, Cat #64252) for 10 minutes. The tissue was counterstained with Haematoxylin (Gatt-Koller Cat #401296170) for 1 minute and mounted with embedding medium. Images were acquired using CellSens Standard software and an Olympus BX53 light microscope with an Olympus UC90 camera. Per slide, images of 5-10 different areas were taken and quantified with Qupath software (49).

### 2.4 Fluorescence assisted cell sorting (FACS)

FACS was performed to quantify the cell populations expressing M1 and M2 polarization markers of HBCs. On the fifth day after isolation, cells were harvested using accutase (Thermo Fischer Scientific, Cat #00-4555-56**)** and gentle scraping. Viability and number of cells after scraping was determined using a CASY cell counter model TT (Innovatis, Bielefeld). At least 1x10^5^ viable cells per tube were used for the experiment. Cells were resuspended in 3% FCS - HBSS solution for 10 min at room temperature to block Fc-receptors and reduce non-specific binding. For surface staining, cells were incubated with a fluorochrome-conjugated antibody in the amount indicated in the **Supplementary Table 1** for 20 minutes at 4°C in the dark. Cells were washed with staining buffer [PBS containing 0.1% BSA (Sigma Aldrich, Cat #A2153) and 2mM EDTA (Thermo Fischer Scientific, Cat #15575020)], centrifuged at 300 g for 5 minutes and resuspended in 200 µL staining buffer. For detection of surface molecules, a minimum of 10000 live events per sample were counted. In order to identify expression of surface markers, cells were separated by size using forward and size scatter (FSC and SSC, respectively), followed by doublet discrimination. In the next step, cells were discriminated into live and dead cells using the 7-AAD dye (BD Biosciences, Cat #559925) by plotting it against the SSC area. In the fourth step cells were plotted for the respective marker against the SSC area. For staining of intracellular molecules, cells were fixed and permeabilized with BD Cytofix/Cytoperm kit (BD Biosciences, Cat #554714). Staining was performed according to the manufacturer’s instructions. A minimum of 10000 events per sample were counted. To investigate the expression of intracellular polarization markers cells were separated by size using forward and size scatter (FSC and SSC, respectively), followed by doublet discrimination and gating against SSC-area and respective marker. The same gating strategy was employed on CTR and PE macrophages. Surface molecules were compensated by individual staining on OneComp eBeadsTM Compensation Beads (Thermo Fischer Scientific, Cat #01-1111-42). Isotype controls corresponding to each fluorochrome in the experiment were used to detect non-specific positive signals. Antibodies used for FACS analysis and their corresponding dilutions are listed in the **Supplementary Table 1**. Cell sorting was performed using a CytoFLEX flow cytometer (Beckman Coulter, Brea, CA, USA) and analysed with FlowJoTM v10.8 software for gate setting and data analysis.

### 2.5 Multiplex ELISA-on-bead Assay

Inflammation 20-Plex Human ProcartaPlex™ Panel (Invitrogen, Thermo Fischer Scientific, Cat #EPX200-12185-901) was used to quantify the secretion of pro- and anti-inflammatory molecules. Human TIMP Magnetic Luminex Performance Assay 4- Plex Kit (R&D Systems, Cat #LKTM003) was used to analyse TIMP secretion. HBCs were cultured in MaM for 5-6 days before supernatants were collected and centrifuged at 4000 rpm, 4°C for 15 minutes. MaM medium processed under the same conditions as the samples served as a blank. Multiplex assays were performed according to the manufacturer’s instructions. Cytokines reaching the detection limits were normalized to total protein content in the supernatant using the Pierce BCA kit (Thermo Fischer Scientific, Cat #23225).

### 2.6 Enzyme- linked immunosorbent assay (ELISA)

TGF-beta 1 Quantikine ELISA kit for human/mouse/rat/porcine/rabbit (R&D Systems, Cat #DB100B) was used for the detection of TGF-β1. Next, to quantify secretion of IL-8, human IL -8/CXCL8 Quantikine ELISA kit (R&D Systems, Cat #D8000C) was used. Both ELISA kits were performed according to the manufacturers’ instructions. To quantify the amount of secreted TGF-β1 and IL-8, cells were cultured in MaM for 5-6 days before collection of the supernatants. MaM medium processed under the same conditions as the samples, but without cells served as a blank. Cytokine levels were normalized to the total protein content in the supernatant measured with the Pierce BCA kit (Thermo Fischer Scientific, Cat #23225), in order to account for deviating volume concentrations.

### 2.7 Quantitative Real-Time PCR (RT-qPCR)

HBCs were washed twice with ice cold Hanks’ salt balanced solution (HBSS, Thermo Fischer Scientific, Cat #14175-053) and harvested in 700 µL QIazol Lysis Reagent (Quiagen, Cat #79306). Total RNA content was isolated using miRNeasy Mini Kit (Quiagen, Cat #217004). Reverse transcription was performed using 1 µg of RNA and Luna Script RT SuperMix Kit (New England BioLabs, Cat #M3010). For qPCR analysis, SYBR Green Luna Universal qPCR Master Mix (New England BioLabs, Cat #M3003) and CFX-384 Touch Real time PCR detection system (Bio-Rad) were used. Expression of target genes was normalized to the following housekeeping genes (*18S*, *RPL30* and *HPRT*) using 2^(^ΔΔCt^) method. Primer sequences used for qPCR analysis are listed in the **Supplementary Table 2**.

### 2.8 Phagocytosis assay measured with FACS

Phagocytosis Assay Kit (Abcam, Cat #ab234053) was used according to the manufacturer’s instructions. For detection of phagocytic activity 1 x 10^6^ cells/ml were used. On the fifth day post isolation HBCs were treated with zymosan slurry and incubated for 3 hours at 21% oxygen and 37°C. After washing steps, cells were detached using accutase (Thermo Fischer Scientific, Cat #00-4555-56) and careful scraping, followed by a washing step with staining buffer containing PBS with 0.1% BSA (Sigma Aldrich, Cat #A2153) and 2mM EDTA (Thermo Fischer Scientific, Cat #15575020). Afterwards measurements were performed in the FITC channel. Untreated cells served as controls. Cells were separated by size using FSC-A and SSC-A, followed by doublet discrimination gating the area and height of FSC. Lastly, the FITC fluorescent signal was determined using histograms. Cell sorting was performed on a CytoFLEX flow cytometer (Beckman Coulter, Brea, CA, USA) using FlowJoTM v10.8software for gate setting and data analysis.

### 2.9 Phagocytosis assay with high-content confocal screening microscope

Phagocytosis Assay Kit (Abcam, Cat #ab234053) was used according to the manufacturer’s instructions. HBCs were seeded at the density of 0.5 x 10^6^ cells/ml in 24-well black/clear bottom plates. 5 µL of zymosan slurry was added to the cells and incubated at 21% oxygen and 37°C for 3 hours, followed by a washing step. Next, cells were fixed in a plate containing 4% neutral buffered paraformaldehyde solution, followed by an intensive wash step with TBE buffer containing 1x TBE and 0.1% Tween (Thermo Fischer Scientific, Cat #003005). HBCs were incubated with Protein Block (Thermo Fischer Scientific, Cat #TL125HL) for 20 minutes. Cells were then counterstained with CD163 (1:100, Thermo Fischer Scientific, Cat #MA1-82342), diluted to working concentration in Antibody diluent (Agilent, Dako, Cat #S0809) and incubated overnight at 4°C. After serial washing steps, the plate was incubated with the secondary antibody Dylight633 (goat versus mouse 1:200, Thermo Fischer Scientific, Cat #35512) for 2 h at room temperature. To stain the nuclei, the plate was counterstained with DAPI (1:1000, Sigma Aldrich, Cat #D9542) diluted in antibody diluent for 10 min. After intensive washing, 300 µL of PBS was added to each well and stored at 4°C. Image acquisition was performed using a Nikon microscope with the Zyla sCMON camera. All statistical analysis was carried out on 25 different locations per well using 20x magnification. For better visualization of phagocytosis shown images in the **Figures 4C–E** were taken with 40x magnification. The number of FITC labelled beads was counted within the cells positive for CD163 and DAPI staining using Nis Elements viewer version 5.20.01 software. Cell surface area was measured using the measuring tool provided within the software.

### 2.10 Gelatin zymography

HBCs supernatants were collected on the fifth day post isolation. The supernatants were centrifuged at 4000 rpm for 15 minutes at 4°C. Total protein concentration was determined using Pierce BCA kit (Thermo Fischer Scientific, Cat #23225) according to the manufacturer’s guidelines. A total of 15 µg of protein sample was diluted with Tris-Glycine SDS sample buffer (Thermo Fisher Scientific, Cat # LC2676) and loaded onto 10% Tris-Glycine gels containing 0.1% gelatin (Thermo Fisher Scientific, Cat #ZY00105BOX) and separated for 135 min at 125 V, 35 mA. After electrophoresis, the gels were incubated in 1x Zymogram Renaturating buffer (Thermo Fisher Scientific, Cat #LC2670) at room temperature with gentle agitation. Followed by 30 minutes incubation with 1x Zymogram developing buffer (Thermo Fisher Scientific, Cat #LC2671). Fresh developing buffer was added and the gels were stored overnight at 37°C. Next day, gels were stained with Coomassie Brilliant Blue (Sigma Aldrich, Cat #1.15444) for 50 minutes and decolorized in 50% distilled water (Fresenius, Cat #C920928): 40% methanol (Sigma Aldrich, Cat #322415): 10% acetic acid (Roth, Cat #3738.5) solution for 10 minutes. Protease activity appearing as a clear band on the dark background was visualized with the ChemiDoc™Touch Imaging System (Bio-Rad). Band densitometry was determined using Image Lab Software Version 6.1 (Bio-Rad).

### 2.11 Statistical analysis

SPSS (IBM SPSS Statistics version 26) was used for statistical calculations. Next, graphs were generated using Graph Pad Prism 9.3.1 software (GraphPad Software Inc.). To test normal distribution Shapiro-Wilk test was used. Skewed data were transformed using natural logarithm (ln) before applied to statistical analysis and re-transformed for the graphical presentation. To assess statistical significance of the patient characteristics (**Table 1**) one-way ANOVA with Tukey’s *post-hoc* test was used. If normality testing failed, Kruskal-Wallis test with Dunn’s *post-hoc* test was performed. Next, to compare differences between three groups (CTR; EO-PE and LO-PE) ANCOVA with adjustment for gestational age and Sidak’s *post-hoc* test was used. Equal variances of variables were verified by Levene´s test. When comparing the effect of HBCs conditioned medium on fpECs without adjustment for gestational age (**Supplementary Figure 5**) two-way ANOVA with Sidak’s *post-hoc* test was used. All values are given as mean ± S.E.M. p-values ≤ 0.05 were considered statistically significant.

## 3 Results

### 3.1 The number and morphology of HBCs is affected by PE

The characteristics of the study population are shown in **Table 1**. Women who developed early (EO, n=8) and late onset (LO, n=6) PE were included in this pilot study. Advanced maternal age, high pre-pregnancy BMI, nulliparity, gestational diabetes, chronic hypertension are some of the risk factors for the development of PE (1). Women in PE groups were significantly older as those in CTR group and their pre-pregnancy BMI ranged from 19.6 to 25.4 kg/m2. As expected, systolic and diastolic blood pressure levels differed significantly between the CTR and PE groups. Gestational age of EO-PE group was significantly lower than of CTR and LO-PE group. Consequently, early gestational age of the EO-PE group is directly related to placental- and fetal weight, both of which were significantly lower than in CTRs. Since development of the placenta depends on gestational age (50), we adjusted the (normally distributed) data for that respective factor. We found a significant difference in the levels of sFlt between EO- and LO-PE group, while there were no significant differences between other clinical parameters (PlGF, platelets, uric acid, AST and ALT).

**Figure 1:**
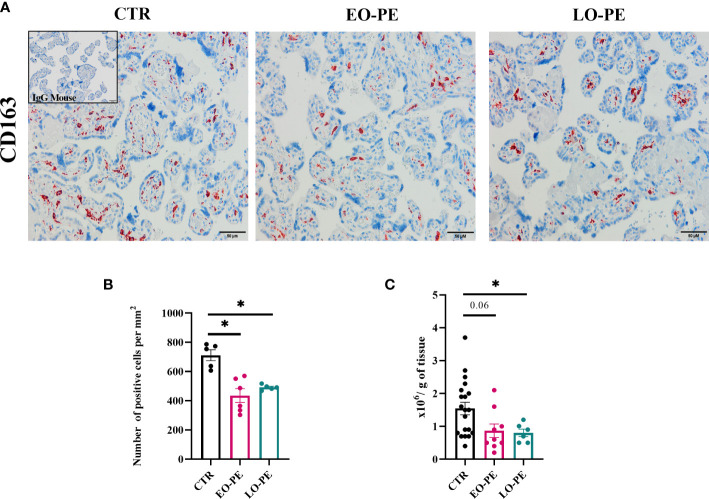
Immunohistochemical assessment of CD163 in placental tissue. **(A)** Representative images of serial sections of CTR (n=5), EO- (n=6) and LO-PE (n=6) are shown. Insert in the top right corner of CTR image depicts negative control stainings (blue). Images were taken at 20x magnification, scale bar represents 50µm. **(B)** Quantification of cells positive for CD163 in CTR, EO- and LO-PE group. Cells were quantified by using Quapath software. **(C)** Average yield of HBCs, adjusted to the wet weight (in grams) of minced tissue for each respective isolation, CTR (n=22), EO-PE (n=8), LO-PE (n=6). Data on **(B)** in **(C)** graphs are presented as mean ± S.E.M, ANCOVA with adjustment for gestational age, followed by Sidak’s *post-hoc* test to examine statistical significance, *p ≤ 0.05.

To study polarization, we first examined the distribution and number of HBCs in placental tissue using immunohistochemistry approach. Since HBCs have been shown to be strongly positive for CD163, we stained 5 µm serial sections of CTR (n=5), EO-PE (n=6) and LO-PE (n=6) placental tissue, mouse IgG served as a negative control. CD163 is used as a marker used for placenta resident macrophages, and if combined with other markers (e.g. Folate receptor-β, CD206, CD209) is often associated with M2 polarization (26, 30, 31, 51). HBCs positive for CD163 were found in the villous stroma, moreover in stem, intermediate and terminal villi of CTR, EO- and LO- PE placentae (**Figure 1A**). Furthermore, quantification of CD163-positive cells revealed a significantly decreased number of stained (**Figure 1B**) cells per mm^2^ in both PE groups, indicating reduced number of HBCs in respective groups. Next, we adjusted the number of isolated HBCs to the wet weight (grams) of placental tissue used for isolation. Consistent with immunohistochemical analysis, we found significantly reduced number of primary LO-PE HBCs. Reduction of HBCs was also observed in the EO-PE group, however, did not reach significance compared to the CTRs (**Figure 1C**).

*In vitro*, cell morphology of HBCs isolated from CTR, EO- and LO-PE placentae showed substantial differences (**Supplementary Figure S1**). Normally, directly after isolation, HBCs are round shaped cells with many vacuoles in the cytosol. Within 48-72 hours after isolation, cells differentiate and develop different shapes. Usually, M2 characterized macrophages exhibit an elongated, spindle-shaped morphology, whereas M1 polarized cells form a round, dendritic cell-like morphology with large filopodia (52, 53). CTR HBCs developed typical M2 features (**Supplementary Figure 1A**), whereas within isolations of EO- and LO-PE HBCs more of round shaped cells with larger filopodia next to M2 morphologies were found (**Supplementary Figures 1B, C).**

### 3.2 EO- and LO-PE HBCs are characterized by different expression of polarization markers

Basal expression of surface and intracellular M1 and M2 markers was determined by FACS (**Tables 2** and **3**) on primary isolated CTR (n=12), EO-PE (n=6) and LO-PE (n=5) HBCs. The gating strategy is shown in **Supplementary Figure 2**. FACS analysis revealed distinct expression of M1 and M2 markers in EO- and LO- PE HBCs (**Table 2**). EO-PE HBCs tend to express higher levels of pro-inflammatory CD11b, CD11c, CD40 (p=0.09) and TLR4 (p ≤ 0.05), whereas expression of listed markers was similarly distributed between LO-PE and CTR HBCs, except for CD209 (p ≤ 0.001). Furthermore, we found significant increase of the major histocompatibility class (MHC) II molecule HLA-DR within the EO-PE group compared to the CTR group (p≤ 0.001). Interestingly, the HLA-DR expression was significantly different between EO- and LO-PE HBCs (p≤ 0.01) as well. Among the M1 markers only expression of CD80 (p=0.05) and TLR1 (p ≤ 0.05) were elevated in LO-PE group. Notably, expression of CD80 and TLR1 was elevated in EO-PE group as well, but only by trend. Interestingly, surface expression of TLR2 was reduced in both EO- (p ≤ 0.05) and LO-PE group. Next, we investigated the expression levels of CD86. Since CD86 can serve as M1 or M2b marker (54, 55), the secretion profile and expression of other markers should be taken into account when interpreting its expression. We found significant induction of the expression of the respective marker in EO-PE HBCs (p ≤ 0.05) compared to the CTR (p ≤ 0.05) or LO-PE (p ≤ 0.05) group. In LO-PE HBCs the expression of CD86 did not differ from the control. In contrast to the increase of pro-inflammatory markers in EO-PE HBCs, a slight decrease in the anti-inflammatory markers CD206 and folate receptor β (FR-β) was observed. Expression pattern of CD206 and FR-β was similar in LO-PE HBCs as well. Expression of CD163 on primary isolated HBCs was evenly distributed between all investigated groups, confirming that CD163 can be used rather as a reliable tissue resident marker (**Figures 1A, B**) than a direct indicator of M2 phenotype. Next, M2 marker CD209 was suppressed in the LO-PE group (p ≤ 0.001). Its expression was only minimally decreased by 10% in EO-PE HBCs group. Interestingly, we found a significant difference in the expression of CD209 between EO- and LO-PE HBCs (p ≤ 0.01).

**Table 2:** Percentage of live cells positive for respective surface polarization markers within the CTR (n=12), EO- (n=6) and LO-PE (n=5) groups.

Surface polarization marker	CTR (% of live cells)	EO-PE (% of live cells)	LO-PE (% of live cells)
CD11B	22.2 ± 9.6	38.7 ± 16.5	28.8 ± 17.0
CD11C	45.1 ± 14.3	69.9 ± 17.8	51.9 ± 11.8
CD40	17.1 ± 5.9	38.9 ± 12.6	22.6 ± 10.9
CD80	2.9 ± 1.8	6.5 ± 3.5	10.5 ± 8.9
CD86	25.0 ± 10.3 *	41.7 ± 3.6 ‡	21.6 ± 6.8
CD163	89.4 ± 3.9	82.0 ± 4.5	81.8 ± 6.8
CD206	47.6 ± 9.8	33.5 ± 6.5	38.2 ± 13.3
CD209	53.3 ± 6.9 †††	43.7± 12.1 ‡‡	22.1 ± 5.2
TLR1	5. 9 ± 6.2 †	9.5 ± 6.2	18.8 ± 13.6
TLR2	6.1 ± 3.9 *	1.2 ± 1.5	5.4 ± 4.5
TLR4	37.3 ± 12.3 *	79.7 ± 19.9	54.2 ± 18.8
HLA-DR	33.8 ± 14.1 ***	77.9 ± 12.6 ‡‡	44.3 ± 18.7
FR-β	79.2 ± 10.0	77.6 ± 20.7	68.4 ± 15.3

Data are presented as mean ± SD. Statistical significance is represented with *p ≤ 0.05, ***p ≤ 0.001; whereas *CTR vs EO-PE; †p ≤ 0.05, †††p ≤ 0.001; whereas †CTR vs LO-PE; ‡ p ≤ 0.05, ‡‡p ≤ 0.01; whereas ‡EO-PE vs LO-PE, by ANCOVA with Sidak’s post-hoc test and adjustment for gestational age.

**Table 3:** Percentage of positive CTR (n=12), EO-PE (n=5) and LO-PE (n=4) HBCs for respective intracellular polarization markers.

Intracellular polarization marker	CTR (% of positive cells)	EO-PE (% of positive cells)	LO-PE (% of positive cells)
CD68	84.0 ± 9.8	70.4 ± 9.1	87.5 ± 5.4
IRF4	91.6 ± 8.0	85.7 ± 11.8	96.2 ± 2.0
IRF5	73.1 ± 12.8	91.5 ± 10.8	85.2 ± 12.0

Data are presented as mean ± SD. Statistical significance was tested by ANCOVA with Sidak’s post-hoc test and adjustment for gestational age.

In addition to the investigation of surface polarization markers, we studied intracellular markers (**Table 3**). Next to the pan-macrophage marker CD68, two important regulators of TLR-Myd88 signaling (56, 57), IRF4 and IRF5 were investigated. Noteworthy, IRF4/IRF5 axis is involved in the initiation control of a specific M1/M2 polarization program. IRF5 as a positive regulator of Myd88 induces the expression of pro-inflammatory genes and establishment of M1 phenotype (58). Whereas IRF4, as a negative regulator of Myd88, leads to the activation of anti-inflammatory genes and initiation of M2 polarization (56, 59).We found the highest expression of IRF5 in EO-PE group and the lowest in CTRs. Expression of IRF4 the regulator of M2 polarization was evenly distributed between CTR and LO-PE group. Importantly, expression of IRF4 in EO-PE HBCs was reduced. Moreover, balance in favour of IRF5 together with higher expression of other surface M1 markers (**Table 2**) indicates possible phenotypic switch towards M1 polarization of EO-PE HBCs.

### 3.3 HBC secretion profile of cytokines and adhesion molecules differs in PE

**Figure 2:**
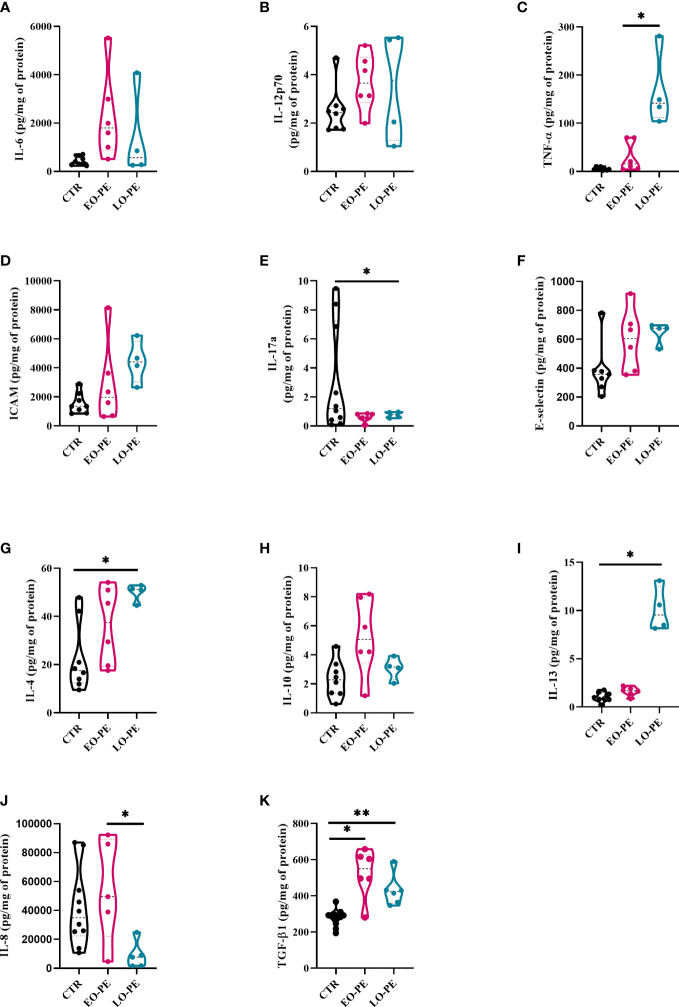
Secretion of cytokines and adhesion molecules by CTR (n=8), EO- (n=6) and LO-PE (n=4) HBCs. Multiplex-ELISA-on-beads assay for followed pro-inflammatory and anti-inflammatory cytokines **(A)** IL-6, **(B)** IL-12p-70, **(C)** TNF-α, **(D)** ICAM, **(E)** IL-17a, **(F)** E-selectin, **(G)** IL-4, **(H)** IL-10 and **(I)** IL-13, respectively. Multiplex was performed in duplicates. **(J)** ELISA against IL-8 performed in duplicates (CTR n=10, EO-PE n=5 and LO-PE n=5). **(K)** ELISA assay against TGF-β1 performed in duplicates (CTR n=12, EO-PE n=6 and LO-PE n=6). Secretion of respective cytokines was normalized to the total protein content measured in the cell culture supernatants. Statistical significance was tested using ANCOVA with adjustment for gestational age followed by Sidak’s *post-hoc* test. *p ≤ 0.05, **p ≤0.01.

Polarized macrophages are known to secrete specific patterns of cytokines, chemokines, and growth factors, allowing us to characterize polarization states (60). Using multiplex ELISA-on-bead technology, we determined the secretion profile of cytokines and chemokines secreted by CTR (n=8), EO-PE (n=6) and LO-PE (n=4) HBCs (**Figure 2**, **Supplementary Figure 3**). Moreover, secretion of IL-8 was determined using ELISA, since its secretion excided the detection limit of the multiplex ELISA (CTR n=10, EO-PE n=5, LO-PE n= 5; **Figure 2J**). Notably, TGF-β1 was measured with ELISA (CTR n=12, EO-PE n=6, LO-PE n=6, **Figure 2K**) since the sample preparation requires acidification of the samples for binding of TGF-β epitopes.

Secretion profile of EO- and LO-PE HBCs differs from the CTR group. First, among pro-inflammatory cytokines, we discovered a trend of an increased secretion of IL-6, IL-12p70 and P-Selectin by EO-PE HBCs (**Figures 2A, B**; **Supplementary Figure 3A**). Contrary, to the EO-PE HBCs where secretion of TNF-α was unchanged, LO-PE released higher amounts of TNF-α as CTRs or EO-PE HBCs (p ≤ 0.05) (**Figure 2C**). LO-PE HBCs secreted higher amounts of ICAM, but only by trend (**Figure 2D**). Moreover, both PE groups secreted less IL-17a compared to CTRs (**Figure 2E**). Although, release of E-Selectin was higher in both PE groups, we did not find any differences in the production of other pro-inflammatory cytokines, such as: IL-1α, IL-1 β, CCL-3, CCL-4, IFN- α, and IFN-γ between investigated groups (**Figure 2F**, **Supplementary Figures 3B–H**). A decrease of IL-8, measured by ELISA was detected in LO-PE HBC group, whereas unchanged between CTR HBCs and EO-PE group. Interestingly, we found a significant difference in the release of IL-8 between EO- and LO-PE HBCs (p ≤ 0.05) (**Figure 2J**).

Next, we examined the secretion of anti-inflammatory cytokines namely, IL-4, IL-10, IL-13, and TGF-β1, which serve as important drivers of M2 polarization (53, 61). We did not find any differences in the secretion of IL-13; however, secretion of IL-4 and IL-10 was increased, but only by trend in EO-PE HBCs. LO-PE HBCs secreted significantly higher levels of IL-4 (p ≤ 0.05) and IL-13 (p ≤ 0.05) compared to CTR HBCs (**Figures 2G–I**). Interestingly, both PE groups released significantly higher amounts of anti-inflammatory TGF-β1, which was even more pronounced in the LO-PE group (p ≤ 0.01) (**Figure 2K**).

Noteworthy, some of the secreted pro-inflammatory cytokines are not reliable identifiers of a specific phenotype, since they are expressed by both M1 and M2 macrophages. E.g. IL-6 is a pro-inflammatory cytokine produced by both M1- and M2a-polarized macrophages (62). In addition, secretion of ICAM is mediated by NF-κB, but can serve as both an M1 and M2 cytokine due to its pro-angiogenic nature (63). The observed changes in the secretion profile indicate a switch in the phenotype and possible protective mechanisms of PE HBCs in an attempt to reduce the extent of inflammation by increasing the production of anti-inflammatory cytokines.

### 3.4 Preeclampsia triggers transcriptional changes of HBC-genes involved in inflammation

**Figure 3:**
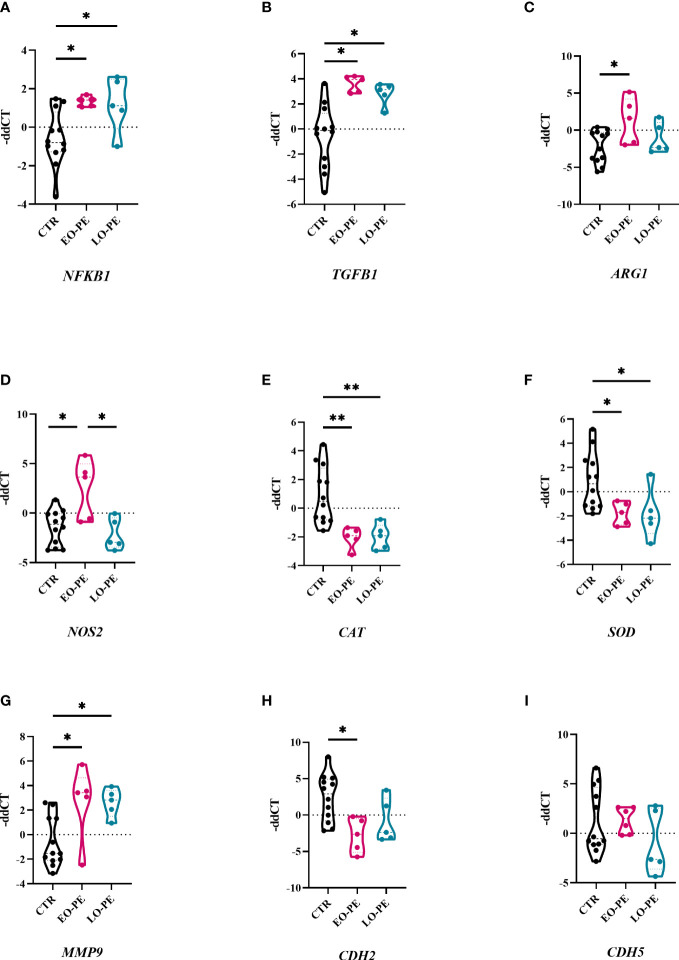
Preeclampsia alters inflammatory related gene expression in HBCs. **(A)** Total RNA of cultivated HBCs was harvested on the fifth day post isolation and analysed by RT-qPCR. **(A)** *NFKB1*, **(B)** *TGFB1*, **(C)** *ARG1*, **(D)** *NOS2*, **(E)** *CAT*, **(F)** *SOD*, **(G)** *MMP9*, **(H)** *CDH2*, **(I)** *CDH5*. In total 12 CTR, 5 EO-PE and 5 LO-PE HBCs isolations in three technical replicates were used. Expression of target genes was normalized to the following housekeeping genes (*18S*, *RPL30* and *HPRT1*) using 2^ΔΔCt^ method. Statistical significance was tested using ANCOVA with adjustment for gestational age followed by Sidak’s *post-hoc* test. *p ≤ 0.05 and **p ≤ 0.01.

Macrophages are capable of responding to the local stimuli and acquiring different phenotypes and functions to meet changing physiological needs (64). Next, we examined transcriptional changes in HBCs that may be triggered by PE. Basal gene expression of CTR (n=12), EO- (n=5) and LO-PE (n=5) HBCs was determined on the fifth day after isolation. We analysed selected genes associated with phenotype and functionality of macrophages undergoing inflammation (**Figure 3**). Dynamic changes in gene expression were observed between the PE subgroups EO and LO. First, we found -as expected - an upregulation of *NFKB1* in both EO- and LO-PE groups (p ≤ 0.05) (**Figure 3A**). Among the inflammatory pathways involved in M1 polarization, NF-κB plays an important role and regulates the expression of pro-inflammatory genes such as cytokines, adhesion molecules and growth factors (65). Next, the expression of *HIF1*, another M1-associated gene was upregulated in the LO-PE group (p ≤ 0.05), whereas its expression was surprisingly decreased in EO-PE group (p ≤ 0.05) (**Supplementary Figure 4**). In line with the increased secretion of *ICAM* (**Figure 2H**), mRNA of the respective gene was upregulated in the both PE groups (**Supplementary Figure 4**). In contrast to the expression of *ICAM, VCAM* was downregulated in EO-PE, whereas in LO-PE group remained on the level of CTRs (**Supplementary Figure 4**). Consistent with secretion of IL-8 (**Figure 2J**), higher fold change of *IL8* was detected in CTR group (**Supplementary Figure 4**). The expression of *TGFB1*, which acts as important M2 inducer (66), was significantly elevated in both, EO- and LO-PE HBCs (p ≤ 0.05) (**Figure 3B**). Although, the differences in the secretion of CCL-4 (**Supplementary Figure 3H**) were not noticeable, higher expression was detected in the CTR group by RT-qPCR. In contrast to the secretion profile of IL -6 and IL -10, which was higher in the PE group (**Figures 2A, H**), qPCR analysis revealed downregulation of respective cytokines in the EO- and LO-PE groups (**Supplementary Figure 4**), possibly due to the tight post-transcriptional gene regulation of these cytokines in particular (67). As polarized macrophages metabolise L-arginine differently, M2 *via* arginase-1 and M1 macrophages *via* nitric oxide synthase (iNOS) (68), we looked at expression of *ARG1* gene, encoding arginase-1, and *NOS2*, encoding iNOS (68). Interestingly, *ARG1* expression was increased in both PE groups (**Figure 3C**). However, expression of *NOS2* was strongly upregulated in EO-PE (p ≤ 0.05); whereas expression of the respective gene in LO-PE HBCs was even lower as in CTR HBCs (**Figure 3D**). In addition to the metabolism of L-arginine, regulation of reactive oxygen species (ROS), represents an important link between M1 and M2 polarization (69). M1 macrophages produce higher amounts of ROS and consequently downregulate antioxidant enzymes such as *CAT* encoding catalase, or *SOD* encoding superoxide dismutase (70), whereas, M2 macrophages are thought to produce lower levels of ROS and express higher levels of *CAT* or *SOD* (71). The expression of the genes *CAT* and *SOD*, was significantly attenuated in both, EO- and LO-PE groups (**Figure 3E, F**). Next in respect to observed TGF-β1 differences, we looked at the expression of genes involved in tissue remodeling and adhesion. Importantly, we found upregulation of *MMP*9 in both PE groups (**Figure 3G**). The expression of other genes involved in tissue remodeling (*MMP2, MMP12, TIMP1, TIMP2*), did not differ between groups (**Supplementary Figure 4**). Adhesion molecules, such as *CDH2* has been downregulated in both EO-PE (p ≤ 0.05) and LO-PE HBCs (**Figure 3H**). Similarly, as *CDH2* we identified reduced expression of *CDH5* in both, EO- and LO- PE HBCs (**Figure 3I**).

In the healthy placenta, HBCs are often found in close proximity to feto-placental endothelial cells (fpEC), and M2 macrophages have the ability to regulate placental angiogenesis by secretion of pro-angiogenic factors (26). To gain insight into their role in angiogenesis, HBCs were examined for the expression of *FLT, VEGFA, KDR*, and *EGFR*; which were all downregulated in EO- and LO-PE group (**Supplementary Figure 4**).

Furthermore, EO-PE and CTR fpECs were treated with conditioned medium (CM) collected from CTR and EO-PE HBCs. In order to further investigate the influence of HBCs on the fpEC, metabolic activity of fpECs using the MTS assay (**Supplementary Figure 5A**) and the proliferation of the fpEC by incorporation of BrDU (**Supplementary Figure 5B**) were measured. CM of CTR HBCs increased the NAD(P)H dehydrogenase activity of CTR fpEC, whereas PE CM had no effect on the activity of PE fpEC (**Supplementary Figure 5A**). A similar effect was observed when the proliferation of CTR fpEC was measured (**Supplementary Figure 5B**).

### 3.5 Phagocytosis of HBCs is altered in PE

**Figure 4:**
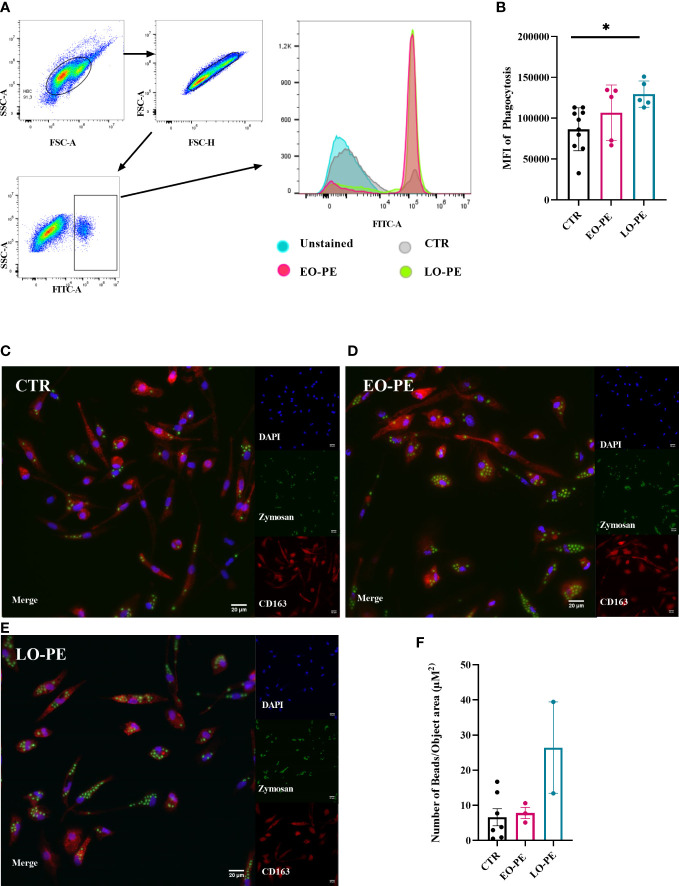
PE HBCs possess a higher phagocytic capacity. **(A)** Gating strategy cells for the measurement of phagocytosis. Cells were separated by size using forward and size scatter (FSC and SSC, respectively), followed by doublet discrimination and gating against SSC-area and FITC -A fluorescent signal. Histograms of one representative experiment are shown, in total 10 CTR, 5 EO-PE and 5 LO-PE HBCs were used. **(B)** Quantification of median fluorescence intensity (MFI) of phagocytosis measured with FACS. **(C)** Visualization of phagocytosis with high content screening microscopy (HCS). HBCs were treated with zymosan beads (green) and co-stained with CD163 (red), DAPI was used to stain nuclei. Representative images of individual experiments are shown. To visualize phagocytosis CTR (**C**, n=7), EO-PE (**D**, n=3) and LO-PE (**E**, n=2) isolations were used. Scale bar represents 20µM. **(F)** Quantification of the phagocytosis measured with high content screening. Analysis was carried out with NisViewer Software, analyzing the number of beads within the CD163 positive HBC cell. All data in **(B, F)** are presented as mean ± S.E.M, ANCOVA with adjustment for gestational age with Sidak’s *post-hoc* test was used for to test statistical significance. *p ≤ 0.05.

Macrophages, as professional phagocytes eliminate pathogens and apoptotic cells. The elimination of apoptotic cells plays an important regulatory role regarding the reduction of the inflammatory burden (72). Phagocytosis was measured and visualised using two different approaches. First, it was assessed by FACS (CTR n=10, EO-PE n=5, LO-PE n=5), where median fluorescence intensity (MFI) was used to quantify the phagocytic activity. PE HBC showed significantly higher phagocytic activity (p ≤ 0.01) than CTRs (**Figures 4A, B**). Second, we analysed phagocytic activity using HCS (**Figures 4C–E**). For better visualisation cells were stained with HBCs tissue resident marker CD163. Analysis of CTR (n=7) and PE (n=5, EO n=3, LO n=2) confirmed higher (though not significant) phagocytosis of PE HBCs (**Figure 4F**). Furthermore, visualisation of phagocytosis allowed us to analyse morphology of the cells, calculating cell size - surface area (µM^2^), which was lower in EO- and LO-PE groups, when compared to the surface area (µM^2^) of CTRs (**Supplementary Figure 6**) confirming *in vitro* observations of smaller round cell morphologies of PE HBC (**Supplementary Figures 1A–C**).

### 3.6 PE attenuates MMP-9 activity of HBCs

**Figure 5:**
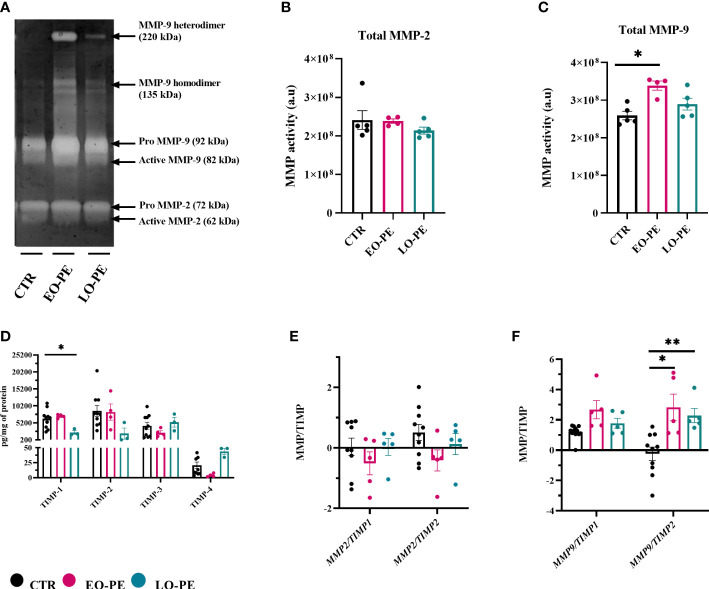
Gelatin zymography demonstrating gelatinolytic activity of HBCs. **(A)** A representative zymogram gel showing MMP-9 and MMP-2 activity of CTR (n=5), EO- (n=4) and LO-PE (n=5) HBCs. Activity was examined in the supernatants collected from CTR and PE HBCs on the 5th day post isolation. In total 15µg of protein measured in supernatants were loaded onto gelatin gels, respectively. To generate the comparable gelatinolytic bands, gelatin zymography was repeated twice. White bands of gelatinase activity observed at 92 and 82kDa are pro- and active form of MMP-9. Moreover, white bands at the size of 72 and 62kDa represents pro- and active form of MMP-2. Quantification of total (pro and active) form of MMP-9 **(B)** and MMP-2 **(C)** was performed as densitometric analysis of respective bands. **(D)** TIMP-1, TIMP-2, TIMP-3 and TIMP-4 levels measured in the cell culture supernatants using multiplex assay. In total CTR (n=10), EO- (n=4) and LO-PE (n=3) HBCs were used in technical duplicates. Secretion of respective TIMPs was normalized to the total protein content measured in the cell culture supernatants. **(E)** mRNA ratio between *MMP2* and *TIMP1* and *TIMP2*. **(F)** mRNA ratio between *MMP9* and *TIMP1* and *TIMP2*. mRNA ratio was calculated from the -ddCT values normalized to the corresponding housekeeping genes (*18S*, *RPL30* and *HRPT1*). The results are showed as a mean ± S.E.M of technical triplicates. Statistical significance was assessed using ANCOVA with adjustment for gestational age followed by Sidak’s *post-hoc* test. *p ≤ 0.05. **p ≤ 0.01.

In a normal placenta M2-polarized HBCs contribute to tissue remodeling and repair (73). To confirm strong upregulation of *MMP9* (**Figure 3G**), we additionally performed gelatin zymography to assess the activity of MMP-2 and MMP-9 (**Figure 5A**). As shown with *MMP2* mRNA expression (**Supplementary Figure 4**), detectable MMP-2 activity (**Figure 5A**) did not differ between studied groups (**Figure 5B**). In line with upregulation of *MMP9*, EO-PE HBCs displayed significantly (p ≤ 0.05) higher MMP-9 activity as CTR. LO-PE HBCs MMP-9 activity was increased, but only by trend (**Figure 5C**).

Expression and production of MMPs are usually tightly regulated within the complex network of their four different tissue inhibitors of metalloproteinases 1-4 (TIMP). HBCs secretion of TIMP- (1, 2, 74, 75) was assessed using a multiplex ELISA-on-bead assay (**Figure 5D**). Production of TIMP-1, TIMP-2, TIMP-3 was unchanged in EO-PE group, we noticed a decreased production of TIMP-4 in the respective group. Furthermore, LO-PE HBCs secretion of TIMP-1 was significantly decreased, followed by trend in the reduced production of TIMP-2. Interestingly, release of TIMP-3 in LO-PE group was similar as in CTR. LO-PE HBCs production of TIMP-4 was elevated, but only by trend. To further explore the gelatinolytic activity of HBCs, the ratio between mRNA expression of *MMP2/TIMP1*, *MMP2/TIMP2*, *MMP9/TIMP1*, and *MMP9/TIMP2* was calculated. Normally, TIMPs regulate inhibition of MMPs by binding in 1:1 reversible complex with the MMPs (76, 77). The shift in MMP/TIMP balance in favor of MMPs reflects as increased extracellular matrix (ECM) proteolysis, or if in favor of TIMP as decreased proteolysis and protection of the ECM (78–80). In CTR, EO- and LO- PE HBCs the ratio between *MMP2* and *TIMP1* or *TIMP2* (**Figure 5E**) stayed unchanged. Similar *MMP9/TIMP1* ratio of investigated groups, was favoring the gelatinolytic activity, indicating that MMP-9 is the main gelatinase produced by HBCs. However, significant differences were demonstrated in *MMP-9/TIMP-2* of EO- and LO-PE HBCs, confirming higher gelatinolytic activity shown with gelatin zymography (**Figures 5A, C, F**).

**Figure 6:**
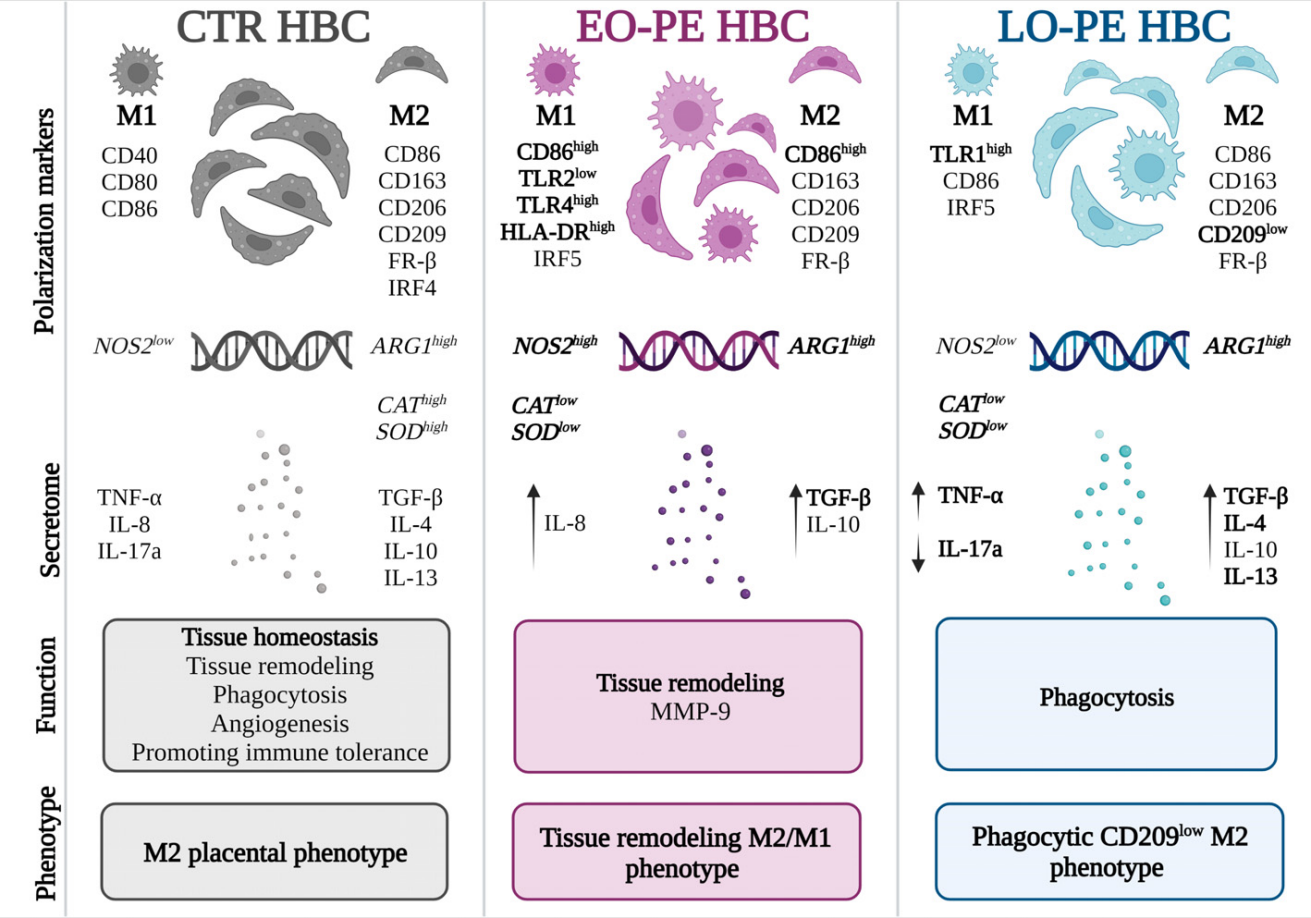
Different polarization patterns of CTR, EO - and LO-PE HBCs. HBCs isolated from CTR placenta develop a specific placental phenotype and express both M1 and M2 polarization markers. In CTR placenta, their M2 nature is reflected by increased expression of *ARG1*, *CAT*, and *SOD*, where HBCs promote tissue repair, angiogenesis, and homeostasis. Our results suggest that EO-PE HBCs develop an M2 phenotype that is strongly shifted toward M1 polarization. Their M2 phenotype is reflected in the upregulation of *ARG1*, secretion of TGF-β, and tissue remodeling function, whereas features of M1 polarization are seen in the increased expression of TLR4, HLA-DR, IRF5, and *NOS2*. In contrast, LO-PE HBCs tend to develop a phagocytic CD209^low^ M2 phenotype with increased production of IL-4, IL-13, and TGF-β. However, the higher expression of TLR1 and increased production of TNF-α indicate a specific pro-inflammatory pattern that differs from the typical M2 polarization. The figure was generated using BioRender. Differences that were significant different between the groups studied are printed in bold.

## 4 Discussion

Hofbauer cells, play a pivotal but also a diverse role in placental physiology by maintaining tissue homeostasis and the tolerogenic environment (26, 36, 41, 81). HBCs plasticity is well characterized by an unique phenotype expressing both, M2 and M1 polarization markers (26, 30, 31). Preeclampsia is an inflammatory condition, accompanied by activation of both the innate and adaptive immune system. These alterations may directly influence the phenotype of HBCs and contribute to placental dysfunction (82). Due to the presumed distinct pathophysiological origins of EO- and LO-PE and the resulting different inflammatory burden within the PE placenta (5, 8, 9, 83), we aimed to determine the phenotypic and functional alterations between different HBCs. One of the more significant findings to emerge from this study is that both, EO- and LO-PE HBCs maintain a profound anti-inflammatory phenotype in the human placenta. In addition to HBCs general adaptive response to inflammatory stimuli, EO-PE HBCs cope differently with signals from their microenvironment.

The EO-PE placenta has been linked to placental malperfusion (84), leading to oxygen deficiency, increased inflammation and oxidative stress which all together deteriorate the mechanisms of placentation early in pregnancy. In contrast, LO-PE placenta has been linked to changes of systemic blood pressure in the mother leading to maternal endothelial dysfunction and oxidative stress, resulting in placental dysfunction (85, 86). Consequently, both subtypes of PE are characterized by excessive inflammation caused by oxidative stress and a hypoxic microenvironment, which affects the appearance and phenotypic composition of immune cells, particularly macrophages (87). To investigate whether the number of HBCs differs between CTR and PE placenta, we first quantified the cells *in situ* using specific markers for tissue-resident macrophages. In agreement, with the findings of Tang et al., where they investigated CD163 positive HBCs in PE placentae (51), we found a decreased number of CD163 - positive macrophages in both EO- and LO-PE placentae. Similarly, Yang et al. observed a significantly reduced number of CD14-positive HBCs in PE (87). In addition, Broekhuizen et al., used combined staining for CD68 and CD163 and observed a significant decrease in double-positive HBCs in EO-PE, whereas the number of double-positive HBCs in LO-PE remained unchanged (18). We verified our immunohistochemical findings by analyzing the yield of primary isolated HBCs *in vitro*. Similar to *in situ*, we obtained a decreased number of primary HBCs isolated from EO- and LO-PE placental tissues. As PE placenta is characterized by an increased number of apoptotic trophoblasts (88, 89), it is likely that HBCs may undergo a similar apoptosis cascade, leading to a decreased number of vital HBCs in culture. According to our results, PE may exacerbate the participation of initial stages of apoptosis in placental tissue which in turn leads to a reduced anti-inflammatory and immunoregulatory capability of remaining HBCs.

Macrophage polarization is a complex process controlled by several factors and mechanisms (90). It is noteworthy that a subpopulation identified with the same markers may have different functions in different tissues and pathologies, adding to the complexity of defining phenotypes (27). At baseline, HBCs express M1 and M2 polarization markers (30, 31). Moreover, HBCs retain alternative M2 polarization in inflammatory complications such as in gestational diabetes mellitus (31), chorioamnionitis (91), or upon *in vitro* stimulation (30). Contrary to expectations, we observed differences in the expression pattern of polarization markers between EO- and LO-PE HBCs. In EO- HBC markers involved in M1 polarization such as TLR4, HLA-DR, CD40, CD80 and CD86 were upregulated, suggesting M1 phenotype. In contrast, in LO-PE HBCs the expression of before mentioned markers was either reduced or similar to CTRs. The anti-inflammatory phenotype of LO-PE HBCs is strongly underpinned by high expression of IRF4, which exerts important function in controlling local cytokine milieu thereby polarization (92). TLR signaling has been proposed as an important link between activation of innate immune system and PE, known to modulate the inflammatory responses (18, 93). Interestingly, Young et al. demonstrated the maintenance of the M2 phenotype of HBCs despite pro-inflammatory treatment and upregulation of TLR4 and increased secretion of pro-inflammatory cytokines (IL-6, IL-8) (37). We demonstrated an upregulation of TLR4 and its downstream mediators *NFKB1* and TNF-α in EO-PE, implying they give up M2 polarization and a shift towards M1. A change of phenotype toward M1 polarization is also suggested by increased expression of IRF5 and *NOS2*. IRF4 and IRF5, both regulators of the Myd88 pathway are crucial for the expression of M1/M2 genes (94). In particular, IRF5 regulates the expression of pro-inflammatory factors such as: TNF-α, IL-6, IL-12p70, CD86 (58). IRF4, on the other hand, is known to compete with IRF5 for interaction with Myd88 to activate the M2 program (56, 92). Its expression has been shown to be induced by anti-inflammatory IL-4 (92, 95). Interestingly, EO-PE HBCs, although favoring IRF5 activation, express IRF4 and consequently secrete both pro- and anti-inflammatory cytokines. A dysbalance of intracellular IRF4 and IRF5 regulating factors may control different phenotypes and the associated contributions to tissue inflammation.

It has been reported that in PE placentae CD163 and FR–β, expression is decreased (51). Although a reduction of CD163 on tissue levels could be confirmed, no differences of CD163 and FR-β expression on the primary isolated HBCs were detected. This inconsistency can be explained because CD163 is used as a tissue-resident marker for placental macrophages rather than only as an M2 marker. Among anti-inflammatory M2 markers, CD209 serves as one of the major M2 markers, moreover, CD209-positive HBCs have been shown to produce IL -10 an immunosuppressive cytokine one of the drivers of immunoregulatory M2 polarization (96). Our results suggest differential expression patterns of CD209 between EO- and LO-PE HBCs. Interestingly, the expression of CD209 was unchanged in CTRs and in the EO-PE group, whereas it was significantly reduced in the LO-PE group. In line, LO-PE HBCs tend to secrete lower levels of IL-10 supporting the notion that this polarization pattern favors regulatory properties of these cells. Yang et al. also reported lower numbers of CD209-positive HBCs in PE placental tissues, but they did not distinguish between onsets of PE (96)

Cytokines are important coordinators that likely promote phenotypic and functional changes of immune cells in inflammation (90, 97). We have shown that both, EO- and LO-PE HBCs produce different regulators of M2 polarization: IL-4, IL-10, IL-13 and TGF-β. Interestingly, whereas in EO-PE an increased production of TGF-β and IL-10 was observed, LO-PE HBCs secreted higher amounts of IL-4, IL-13, and TGF-β. IL-10 and IL-4, have been recognized as inducers of *ARG1*, which represents a hallmark of M2 polarization (97–100) IL-10 is a cytokine produced mainly by M2 macrophages (58) and its anti-inflammatory effect has been demonstrated by reduced production of pro-inflammatory cytokines such as TNF-α, IL-6, and IL-12 (101, 102). Moreover, although pro-inflammatory M1 marker - IRF5 has been shown to inhibit transcription of IL-10 and TGF-β (58, 103), this regulation may be impaired in EO-PE, where basal secretion of IL-10 and TGF-β levels were increased. Enhanced expression of M1 polarization markers, upregulation of both M1 *NOS2* and M2 *ARG1*, accompanied by secretion of anti-inflammatory cytokines, indicate a development of a specific M1 and M2 phenotype of EO-PE HBCs. On the other hand, LO-PE HBCs downregulate *NOS2* and express *ARG1* at the same level as CTRs HBCs, suggesting their anti-inflammatory phenotype.

Phenotypic plasticity enables macrophages to perform a variety of functions required for maintenance of homeostasis and rapid termination of inflammation in their microenvironment (66). The resolution of acute inflammation is a well-orchestrated synergistic process and can be divided into three phases on the way back to cell homeostasis. First, inflammation is downregulated by the temporal switching of secreted lipid mediators, then the clearance of debris and apoptotic cells by phagocytically active macrophages, and finally, tissue repair and angiogenesis are stimulated by the pro-resolving phenotype of macrophages (20, 24, 54, 104). We demonstrated a higher phagocytic capacity of EO- and LO-PE compared to normal HBCs, with concomitantly significantly higher levels of TGF-β produced by both groups. Interestingly, TGF-β in monocytes and macrophages is known for its role in maintaining the resolution of inflammation by increasing phagocytosis and restoring tissue integrity (105–107). The increased phagocytosis and production of anti-inflammatory cytokines (90) may indicate one of the regulatory mechanisms that determine M2 phenotype of EO- and LO-PE HBCs by preventing a direct switch to M1 polarization.

Following clearance of cell-debris, M2 macrophages initiate events that are critical for tissue repair. These include the production of extracellular matrix (ECM), MMPs and the promotion of angiogenesis (108). Furthermore, MMPs make an important contribution to ECM repair (108), and macrophages require active MMP-9 for migration during an inflammatory response (109). TGF-β acts as a regulator of ECM production, reflecting its role in the tissue remodeling (110). We have shown compared to normal HBCs that both EO- and LO-PE HBCs secrete higher levels of MMP-9 and TGF-β. Interestingly, one of the many functions of MMP-9 is also to activate the inactive form of TGF-β (111), suggesting a possible mechanism driving polarization toward M2. MMP-9 is regulated by the expression of TIMPs, which has been shown to have pro-angiogenic features and is unique to M2 macrophages (112). Although the MMP9/TIMP2 ratio of EO- and LO-PE HBCs favors M2 polarization, we have shown that EO-PE HBCs were unable to stimulate proliferation of endothelial cells. Ability to enhance endothelial proliferation and consequently angiogenesis is one of the traits of M2 macrophages in the process of resolution of inflammation (104). The unsuccessful activation of proliferation of endothelial cells reveals another aspect of the pro-inflammatory M1 signature of EO-PE HBCs. It is more likely that they contribute to endothelial dysfunction instead of positive pro-angiogenic endothelial activation, but further investigation is needed.

Knowledge about the function of macrophages in PE is still insufficient. Our study focused on the *in vitro* polarization and functionality of EO- and LO-PE HBCs. Our results suggest that EO-PE HBCs develop a strong M1 signature, but despite the M1 features and PE inflammatory microenvironment, they still attempt to resolve inflammation by upregulating M2 anti-inflammatory factors and functions. Based on the fact that the expression of CD209 in EO-PE HBCs is at basal levels as in CTR HBCs, and expression of CD86 and secretion of TGF-β are increased, they might develop an immunoregulatory M2b and a tissue remodeling M2a phenotype with features of M1 polarization based on the increased expression of TLR4, HLA-DR, and IRF5. In contrast, LO-PE HBCs tend to develop a phagocytic M2 phenotype with increased production of IL-4, IL-13, and TGF-β. However, the higher expression of TLR1, TLR4, and CD80 and increased production of TNF-α indicate a specific pro-inflammatory pattern distinct from typical M2 polarization (**Figure 6**). Given the strength of our study, namely the use of a large number of clinically well-characterized samples from EO- and LO-PE placenta, there are some apparent limitations. First, macrophage polarization is a dynamic process, and therefore the choice of polarization markers included may vary among investigators. Because characterization of macrophage phenotype using polarization markers and cytokine release is rather descriptive, we chose to use functional assays to determine the relevant physiological functions of macrophages in addition to their phenotype. Second, primary HBCs might develop a different polarization pattern than *in vivo* because of the sensitivity of primary HBCs to the *in vitro* environment. However, we and others have shown that Hofbauer cells have a very stable phenotype *in vitro* that is difficult to alter and correlates with the phenotype in tissue *in vivo* (30, 37).

In conclusion, to the best of our knowledge, this is the first study to show a pivotal difference of the polarization pattern between EO - and LO-PE HBCs *in vitro*. We demonstrated that the inflammatory environment of PE causes the phenotypic changes observed between early and late PE HBCs. The changes in polarization patterns indicate different etiologies of PE, as EO-PE is associated with inflammation on the placental side, whereas LO-PE results from a maternal inflammatory response. Furthermore, since placental immune cells responds differently to the source of inflammation, PE could be identified as different entities with a common phenotype rather than a single disorder.

## Data availability statement

The original contributions presented in the study are included in the article/**Supplementary Material**. Further inquiries can be directed to the corresponding author.

## Ethics statement

The studies involving human participants were reviewed and approved by Institutional ethics committee of the Medical University of Graz (29-319 ex 16/17). The patients/participants provided their written informed consent to participate in this study.

## Author contributions

MHM and CW conceived the study and designed the experiments. MHM performed the experiments and analysed the data. Reviewing and editing was done by CS and HF. MHM and CW wrote the original draft manuscript. All authors contributed to the article and approved the submitted version.

## References

[1] Gestational hypertension and preeclampsia: ACOG practice bulletin, number 222. Obstet Gynecol (2020) 135:e237–60. doi: 10.1097/AOG.0000000000003891 32443079

[2] Practice Bulletin No ACOG. 203: Chronic hypertension in pregnancy. Obstet Gynecol (2019) 133:e26–50. doi: 10.1097/AOG.0000000000003020 30575676

[3] Brown MA Magee LA Kenny LC Karumanchi SA McCarthy FP Saito S. Hypertensive disorders of pregnancy. Hypertension (2018) 72:24–43. doi: 10.1161/HYPERTENSIONAHA.117.10803 29899139

[4] Wójtowicz A Zembala-Szczerba M Babczyk D Kołodziejczyk-Pietruszka M Lewaczyńska O Huras H. Early-and late-onset preeclampsia: A comprehensive cohort study of laboratory and clinical findings according to the new ISHHP criteria. Int J Hypertens (2019) 2019:4108271. doi: 10.1155/2019/4108271 31637053 PMC6766116

[5] von Dadelszen P Magee LA Roberts JM. Subclassification of preeclampsia. Hypertens Pregnancy (2003) 22:143–8. doi: 10.1081/PRG-120021060 12908998

[6] Tranquilli AL Brown MA Zeeman GG Dekker G Sibai BM. The definition of severe and early-onset preeclampsia. statements from the international society for the study of hypertension in pregnancy (ISSHP). Pregnancy Hypertension: Int J Women’s Cardiovasc Health (2013) 3:44–7. doi: 10.1016/J.PREGHY.2012.11.001 26105740

[7] Huppertz B. Placental origins of preeclampsia: Challenging the current hypothesis. Hypertension (2008) 51:970–5. doi: 10.1161/HYPERTENSIONAHA.107.107607 18259009

[8] Li XL Guo PL Xue Y Gou WL Tong M Chen Q. An analysis of the differences between early and late preeclampsia with severe hypertension. Pregnancy Hypertension: Int J Women’s Cardiovasc Health (2016) 6:47–52. doi: 10.1016/J.PREGHY.2015.12.003 26955772

[9] Nelson DB Ziadie MS McIntire DD Rogers BB Leveno KJ. Placental pathology suggesting that preeclampsia is more than one disease. Am J Obstet Gynecol (2014) 210:66. doi: 10.1016/J.AJOG.2013.09.010 24036400

[10] Stergiotou I Crispi F Valenzuela-Alcaraz B Bijnens B Gratacos E. Patterns of maternal vascular remodeling and responsiveness in early- versus late-onset preeclampsia. Am J Obstet Gynecol (2013) 209:558. doi: 10.1016/J.AJOG.2013.07.030 23911383

[11] del Gaudio I Sasset L di Lorenzo A Wadsack C. Sphingolipid signature of human feto-placental vasculature in preeclampsia. Int J Mol Sci (2020) 21(3):1019. doi: 10.3390/ijms21031019 32033121 PMC7037072

[12] Murthi P Pinar AA Dimitriadis E Samuel CS. Inflammasomes–a molecular link for altered immunoregulation and inflammation mediated vascular dysfunction in preeclampsia. Int J Mol Sci (2020) 21:1406. doi: 10.3390/IJMS21041406 32093005 PMC7073120

[13] Borzychowski AM Sargent IL Redman CWG. Inflammation and pre-eclampsia. Semin Fetal Neonatal Med (2006) 11:309–16. doi: 10.1016/J.SINY.2006.04.001 16828580

[14] Sharma A Satyam A Sharma JB. Leptin, IL-10 and inflammatory markers (TNF-α, IL-6 and IL-8) in pre-eclamptic, normotensive pregnant and healthy non-pregnant women. Am J Reprod Immunol (2007) 58:21–30. doi: 10.1111/J.1600-0897.2007.00486.X 17565544

[15] Vishnyakova P Poltavets A Nikitina M Muminova K Potapova A Vtorushina V. Preeclampsia: inflammatory signature of decidual cells in early manifestation of disease. Placenta (2021) 104:277–83. doi: 10.1016/j.placenta.2021.01.011 33472135

[16] Aneman I Pienaar D Suvakov S Simic TP Garovic VD McClements L. Mechanisms of key innate immune cells in early- and late-onset preeclampsia. Front Immunol (2020) 11:1864/XML/NLM. doi: 10.3389/FIMMU.2020.01864/XML/NLM 33013837 PMC7462000

[17] Han X Ghaemi MS Ando K Peterson LS Ganio EA Tsai AS. Differential dynamics of the maternal immune system in healthy pregnancy and preeclampsia. Front Immunol (2019) 10:1305/BIBTEX. doi: 10.3389/FIMMU.2019.01305/BIBTEX 31263463 PMC6584811

[18] Broekhuizen M Hitzerd E van den Bosch TPP Dumas J Verdijk RM van Rijn BB. The placental innate immune system is altered in early-onset preeclampsia, but not in late-onset preeclampsia. Front Immunol (2021) 12:780043/BIBTEX. doi: 10.3389/FIMMU.2021.780043/BIBTEX 34992598 PMC8724430

[19] Ma Y Ye Y Zhang J Ruan CC Gao PJ. Immune imbalance is associated with the development of preeclampsia. Med (United States) (2019) 98(14):e15080. doi: 10.1097/MD.0000000000015080 PMC6455976 30946359

[20] Atri C Guerfali FZ Laouini D. Role of human macrophage polarization in inflammation during infectious diseases. Int J Mol Sci (2018) 19(6):1801. doi: 10.3390/IJMS19061801 29921749 PMC6032107

[21] Shapouri-Moghaddam A Mohammadian S Vazini H Taghadosi M Esmaeili SA Mardani F. Macrophage plasticity, polarization, and function in health and disease. J Cell Physiol (2018) 233:6425–40. doi: 10.1002/jcp.26429 PMC13482560 29319160

[22] Xue J Schmidt SV Sander J Draffehn A Krebs W Quester I. Transcriptome-based network analysis reveals a spectrum model of human macrophage activation. Immunity (2014) 40:274. doi: 10.1016/J.IMMUNI.2014.01.006 24530056 PMC3991396

[23] Takiguchi H Yang CX Yang CWT Sahin B Whalen BA Milne S. Macrophages with reduced expressions of classical M1 and M2 surface markers in human bronchoalveolar lavage fluid exhibit pro-inflammatory gene signatures. Sci Rep (2021) 11:1–11. doi: 10.1038/s41598-021-87720-y 33859282 PMC8050093

[24] Porcheray F Viaud S Rimaniol A-C Léone C Samah B Dereuddre-Bosquet N. Macrophage activation switching: an asset for the resolution of inflammation. Clin Exp Immunol (2005) 142:481. doi: 10.1111/J.1365-2249.2005.02934.X 16297160 PMC1809537

[25] Oshi M Tokumaru Y Asaoka M Yan L Satyananda V Matsuyama R. M1 macrophage and M1/M2 ratio defined by transcriptomic signatures resemble only part of their conventional clinical characteristics in breast cancer. Sci Rep (2020) 10:1–12. doi: 10.1038/s41598-020-73624-w 33024179 PMC7538579

[26] Loegl J Hiden U Nussbaumer E Schliefsteiner C Cvitic S Lang I. Hofbauer cells of M2a, M2b and M2c polarization may regulate feto-placental angiogenesis. Reproduction (2016) 152:447–55. doi: 10.1530/REP-16-0159 27534571

[27] Martinez FO Gordon S. The M1 and M2 paradigm of macrophage activation: time for reassessment. F1000Prime Rep (2014) 6:13. doi: 10.12703/P6-13 24669294 PMC3944738

[28] Turco MY Moffett A. Development of the human placenta. Development (2019) 146(22):dev163428. doi: 10.1242/DEV.163428 31776138

[29] Reyes L Wolfe B Golos T. “Hofbauer cells: Placental macrophages of fetal origin”. In: Results Problems Cell Differentiation (2017) 62:45–60. doi: 10.1007/978-3-319-54090-0_3 28455705

[30] Schliefsteiner C Ibesich S Wadsack C. Placental hofbauer cell polarization resists inflammatory cues *In vitro* . Int J Mol Sci (2020) 21(3):736. doi: 10.3390/ijms21030736 31979196 PMC7038058

[31] Schliefsteiner C Peinhaupt M Kopp S Lögl J Lang-Olip I Hiden U. Human placental hofbauer cells maintain an anti-inflammatory M2 phenotype despite the presence of gestational diabetes mellitus. Front Immunol (2017) 8:888. doi: 10.3389/fimmu.2017.00888 28824621 PMC5534476

[32] Tang Z Niven-Fairchild T Tadesse S Norwitz ER Buhimschi CS Buhimschi IA. Glucocorticoids enhance CD163 expression in placental hofbauer cells. Endocrinology (2013) 154:471–82. doi: 10.1210/EN.2012-1575 PMC3529384 23142809

[33] Kim SY Romero R Tarca AL Bhatti G Kim CJ Lee J. Methylome of fetal and maternal monocytes and macrophages at the feto-maternal interface. Am J Reprod Immunol (2012) 68:8–27. doi: 10.1111/J.1600-0897.2012.01108.X 22385097 PMC3479407

[34] Goldstein J Braverman M Salafia C Buckley P. The phenotype of human placental macrophages and its variation with gestational age(1988) (Accessed November 5, 2021). PMC1880826 3264459

[35] Zulu MZ Martinez FO Gordon S Gray CM. The elusive role of placental macrophages: The hofbauer cell. J Innate Immun (2019) 11:447–56. doi: 10.1159/000497416 PMC6758944 30970346

[36] Thomas JR Appios A Zhao X Dutkiewicz R Donde M Lee CYC. Phenotypic and functional characterization of first-trimester human placental macrophages, hofbauer cells. J Exp Med (2020) 218(1):e20200891. doi: 10.1084/JEM.20200891 PMC7579740 33075123

[37] Young OM Tang Z Niven-Fairchild T Tadesse S Krikun G Norwitz ER. Toll-like receptor-mediated responses by placental hofbauer cells (HBCs): A potential pro-inflammatory role for fetal M2 macrophages. Am J Reprod Immunol (2015) 73:22–35. doi: 10.1111/aji.12336 25345551 PMC4268350

[38] Svensson-Arvelund J Mehta RB Lindau R Mirrasekhian E Rodriguez-Martinez H Berg G. The human fetal placenta promotes tolerance against the semiallogeneic fetus by inducing regulatory T cells and homeostatic M2 macrophages. J Immunol (2015) 194:1534–44. doi: 10.4049/JIMMUNOL.1401536 25560409

[39] Cervar M Blaschitz A Dohr G Desoye G. Paracrine regulation of distinct trophoblast functions *in vitro* by placental macrophages. Cell Tissue Res (1999) 295:297–305. doi: 10.1007/S004410051236 9931376

[40] Khan S Katabuchi H Araki M Nishimura R Okamura H. Human villous macrophage-conditioned media enhance human trophoblast growth and differentiation. In Vitro Biol Reprod (2000) 62:1075–83. doi: 10.1095/BIOLREPROD62.4.1075 10727280

[41] Anteby EY Natanson-Yaron S Greenfield C Goldman-Wohl D Haimov-Kochman R Holzer H. Human placental hofbauer cells express sprouty proteins: a possible modulating mechanism of villous branching. Placenta (2005) 26:476–83. doi: 10.1016/J.PLACENTA.2004.08.008 15950061

[42] Seval Y Korgun ET Demir R. Hofbauer cells in early human placenta: possible implications in vasculogenesis and angiogenesis. Placenta (2007) 28:841–5. doi: 10.1016/J.PLACENTA.2007.01.010 17350092

[43] ben Amara A Gorvel L Baulan K Derain-Court J Buffat C Vérollet C. Placental macrophages are impaired in chorioamnionitis, an infectious pathology of the placenta. J Immunol (2013) 191:5501–14. doi: 10.4049/jimmunol.1300988 24163411

[44] Vinnars M-TN Rindsjö E Ghazi S Sundberg A Papadogiannakis N. The number of CD68+ (Hofbauer) cells is decreased in placentas with chorioamnionitis and with advancing gestational age. Pediatr Dev Pathol (2010) 13:300–4. doi: 10.2350/09-03-0632-OA.1 19642814

[45] Toti P Arcuri F Tang Z Schatz F Zambrano E Mor G. Focal increases of fetal macrophages in placentas from pregnancies with histological chorioamnionitis: Potential role of fibroblast monocyte chemotactic protein-1. Am J Reprod Immunol (2011) 65:470–9. doi: 10.1111/j.1600-0897.2010.00927.x PMC3071455 21087336

[46] Kim JS Romero R Kim MR Kim YM Friel L Espinoza J. Involvement of hofbauer cells and maternal T cells in villitis of unknown aetiology. Histopathology (2008) 52:457–64. doi: 10.1111/j.1365-2559.2008.02964.x PMC2896045 18315598

[47] Quicke KM Bowen JR Johnson EL McDonald CE Ma H O’Neal JT. Zika virus infects human placental macrophages. Cell Host Microbe (2016) 20:83–90. doi: 10.1016/J.CHOM.2016.05.015 27247001 PMC5166429

[48] Tang Z Tadesse S Norwitz E Mor G Abrahams VM Guller S. Isolation of hofbauer cells from human term placentas with high yield and purity. Am J Reprod Immunol (2011) 66:336–48. doi: 10.1111/j.1600-0897.2011.01006.x PMC3154981 21545365

[49] Bankhead P Loughrey MB Fernández JA Dombrowski Y McArt DG Dunne PD. QuPath: Open source software for digital pathology image analysis. Sci Rep (2017) 7:1–7. doi: 10.1038/s41598-017-17204-5 29203879 PMC5715110

[50] Moldenhauer JS Stanek J Warshak C Khoury J Sibai B. The frequency and severity of placental findings in women with preeclampsia are gestational age dependent. Am J Obstet Gynecol (2003) 189:1173–7. doi: 10.1067/S0002-9378(03)00576-3 14586374

[51] Tang Z Buhimschi IA Buhimschi CS Tadesse S Norwitz E Niven-Fairchild T. Decreased levels of folate receptor-β and reduced numbers of fetal macrophages (Hofbauer cells) in placentas from pregnancies with severe pre-eclampsia. Am J Reprod Immunol (2013) 70:104–15. doi: 10.1111/AJI.12112 PMC3686834 23480364

[52] McWhorter FY Wang T Nguyen P Chung T Liu WF. Modulation of macrophage phenotype by cell shape. Proc Natl Acad Sci (2013) 110:17253–8. doi: 10.1073/PNAS.1308887110 PMC3808615 24101477

[53] Porta C Riboldi E Ippolito A Sica A. Molecular and epigenetic basis of macrophage polarized activation. Semin Immunol (2015) 27:237–48. doi: 10.1016/J.SMIM.2015.10.003 26561250

[54] Wang Lx Zhang S Wu Hj Rong X Guo J. M2b macrophage polarization and its roles in diseases. J Leukoc Biol (2019) 106:345–58. doi: 10.1002/JLB.3RU1018-378RR PMC7379745 30576000

[55] Martinez FO Sica A Mantovani A Locati M. Macrophage activation and polarization. Front Bioscience (2008) 13:453–61. doi: 10.2741/2692/PDF 17981560

[56] Negishi H Ohba Y Yanai H Takaoka A Honma K Yui K. Negative regulation of toll-like-receptor signaling by IRF-4. Proc Natl Acad Sci (2005) 102:15989–94. doi: 10.1073/PNAS.0508327102 PMC1257749 16236719

[57] Takaoka A Yanai H Kondo S Duncan G Negishi H Mizutani T. Integral role of IRF-5 in the gene induction programme activated by toll-like receptors. Nature (2005) 434:243–9. doi: 10.1038/nature03308 15665823

[58] Krausgruber T Blazek K Smallie T Alzabin S Lockstone H Sahgal N. IRF5 promotes inflammatory macrophage polarization and TH1-TH17 responses. Nat Immunol (2011) 12:231–8. doi: 10.1038/ni.1990 21240265

[59] Honma K Udono H Kohno T Yamamoto K Ogawa A Takemori T. Interferon regulatory factor 4 negatively regulates the production of proinflammatory cytokines by macrophages in response to LPS. Proc Natl Acad Sci (2005) 102:16001–6. doi: 10.1073/PNAS.0504226102 PMC1276050 16243976

[60] Raggi F Pelassa S Pierobon D Penco F Gattorno M Novelli F. Regulation of human macrophage M1–M2 polarization balance by hypoxia and the triggering receptor expressed on myeloid cells-1. Front Immunol (2017) 0:1097. doi: 10.3389/FIMMU.2017.01097 PMC5594076 28936211

[61] Makita N Hizukuri Y Yamashiro K Murakawa M Hayashi Y. IL-10 enhances the phenotype of M2 macrophages induced by IL-4 and confers the ability to increase eosinophil migration. Int Immunol (2015) 27:131–41. doi: 10.1093/INTIMM/DXU090 25267883

[62] Fernando MR Reyes JL Iannuzzi J Leung G McKay DM. The pro-inflammatory cytokine, interleukin-6, enhances the polarization of alternatively activated macrophages. PloS One (2014) 9:e94188. doi: 10.1371/JOURNAL.PONE.0094188 24736635 PMC3988054

[63] Frank PG Lisanti MP. ICAM-1: role in inflammation and in the regulation of vascular permeability. Am J Physiol Heart Circ Physiol (2008) 295:H926. doi: 10.1152/AJPHEART.00779.2008 18689494 PMC2544488

[64] Hu G Su Y Kang BH Fan Z Dong T Brown DR. High-throughput phenotypic screen and transcriptional analysis identify new compounds and targets for macrophage reprogramming. Nat Commun (2021) 12:1–14. doi: 10.1038/s41467-021-21066-x 33536439 PMC7858590

[65] Liu T Zhang L Joo D Sun S-C. NF-κB signaling in inflammation. Signal Transduction Targeted Ther (2017) 2:1–9. doi: 10.1038/sigtrans.2017.23 PMC5661633 29158945

[66] Murray PJ Allen JE Biswas SK Fisher EA Gilroy DW Goerdt S. Macrophage activation and polarization: Nomenclature and experimental guidelines. Immunity (2014) 41:14–20. doi: 10.1016/J.IMMUNI.2014.06.008 25035950 PMC4123412

[67] Palanisamy V Jakymiw A van Tubergen EA D’Silva NJ Kirkwood KL. Control of cytokine mRNA expression by RNA-binding proteins and microRNAs. J Dent Res (2012) 91:651. doi: 10.1177/0022034512437372 22302144 PMC3383846

[68] Rath M Müller I Kropf P Closs EI Munder M. Metabolism *via* arginase or nitric oxide synthase: Two competing arginine pathways in macrophages. Front Immunol (2014) 0:532. doi: 10.3389/FIMMU.2014.00532 PMC4209874 25386178

[69] Zhang Y Choksi S Chen K Pobezinskaya Y Linnoila I Liu Z-G. ROS play a critical role in the differentiation of alternatively activated macrophages and the occurrence of tumor-associated macrophages. Cell Res (2013) 23:898–914. doi: 10.1038/cr.2013.75 23752925 PMC3698641

[70] Rendra E Riabov V Mossel DM Sevastyanova T Harmsen MC Kzhyshkowska J. Reactive oxygen species (ROS) in macrophage activation and function in diabetes. Immunobiology (2019) 224:242–53. doi: 10.1016/J.IMBIO.2018.11.010 30739804

[71] Griess B Mir S Datta K Teoh-Fitzgerald M. Scavenging reactive oxygen species selectively inhibits M2 macrophage polarization and their pro-tumorigenic function in part, *via* Stat3 suppression. Free Radic Biol Med (2020) 147:48–60. doi: 10.1016/J.FREERADBIOMED.2019.12.018 31863907 PMC10035558

[72] Klöditz K Fadeel B. Three cell deaths and a funeral: macrophage clearance of cells undergoing distinct modes of cell death. Cell Death Discov (2019) 5:1–9. doi: 10.1038/s41420-019-0146-x PMC6368547 30774993

[73] Reyes L Golos TG. Hofbauer cells: Their role in healthy and complicated pregnancy. Front Immunol (2018) 9:2628. doi: 10.3389/fimmu.2018.02628 30498493 PMC6249321

[74] Raguema N Moustadraf S Bertagnolli M. Immune and apoptosis mechanisms regulating placental development and vascularization in preeclampsia. Front Physiol (2020) 11:98. doi: 10.3389/fphys.2020.00098 32116801 PMC7026478

[75] Phipps EA Thadhani R Benzing T Karumanchi SA. Pre-eclampsia: pathogenesis, novel diagnostics and therapies. Nat Rev Nephrol (2019) 15:275–89. doi: 10.1038/s41581-019-0119-6 PMC6472952 30792480

[76] Brew K Nagase H. The tissue inhibitors of metalloproteinases (TIMPs): An ancient family with structural and functional diversity. Biochim Biophys Acta (2010) 1803:55. doi: 10.1016/J.BBAMCR.2010.01.003 20080133 PMC2853873

[77] Cataldo DD Gueders M Munaut C Rocks N Bartsch P Foidart J-M. Matrix metalloproteinases and tissue inhibitors of matrix metalloproteinases mRNA transcripts in the bronchial secretions of asthmatics. Lab Invest (2004) 84:418–24. doi: 10.1038/labinvest.3700063 14968124

[78] Visse R Nagase H. Matrix metalloproteinases and tissue inhibitors of metalloproteinases. Circ Res (2003) 92:827–39. doi: 10.1161/01.RES.0000070112.80711.3D 12730128

[79] Gardner K Arnoczky SP Caballero O Lavagnino M. The effect of stress-deprivation and cyclic loading on the TIMP/MMP ratio in tendon cells: An in vitro experimental study. Disabil Rehabil (2008). 30(20–22):1523–9. doi: 10.1080/09638280701785395 18665569

[80] Arpino V Brock M Gill SE. The role of TIMPs in regulation of extracellular matrix proteolysis. Matrix Biol (2015) 44–46:247–54. doi: 10.1016/J.MATBIO.2015.03.005 25805621

[81] Svensson J Jenmalm MC Matussek A Geffers R Berg G Ernerudh J. Macrophages at the fetal–maternal interface express markers of alternative activation and are induced by m-CSF and IL-10. J Immunol (2011) 187:3671–82. doi: 10.4049/JIMMUNOL.1100130 21890660

[82] Rana S Lemoine E Granger J Karumanchi SA. Preeclampsia: Pathophysiology, challenges, and perspectives. Circ Res (2019) 124:1094–112. doi: 10.1161/CIRCRESAHA.118.313276/FORMAT/EPUB 30920918

[83] Ren Z Gao Y Gao Y Liang G Chen Q Jiang S. Distinct placental molecular processes associated with early-onset and late-onset preeclampsia. Theranostics (2021) 11:5028–44. doi: 10.7150/THNO.56141 PMC7978310 33754042

[84] Kovo M Schreiber L Ben-Haroush A Gold E Golan A Bar J. The placental component in early-onset and late-onset preeclampsia in relation to fetal growth restriction. Prenat Diagn (2012) 32:632–7. doi: 10.1002/PD.3872 22565848

[85] Sohlberg S Mulic-Lutvica A Lindgren P Ortiz-Nieto F Wikström AK Wikström J. Placental perfusion in normal pregnancy and early and late preeclampsia: A magnetic resonance imaging study. Placenta (2014) 35:202–6. doi: 10.1016/J.PLACENTA.2014.01.008 24529946

[86] Marín R Chiarello DI Abad C Rojas D Toledo F Sobrevia L. Oxidative stress and mitochondrial dysfunction in early-onset and late-onset preeclampsia. Biochim Biophys Acta (BBA) - Mol Basis Dis (2020) 1866:165961. doi: 10.1016/J.BBADIS.2020.165961 32916282

[87] Saito S Shiozaki A Nakashima A Sakai M Sasaki Y. The role of the immune system in preeclampsia. Mol Aspects Med (2007) 28:192–209. doi: 10.1016/J.MAM.2007.02.006 17433431

[88] DiFederico E Genbacev O Fisher SJ. Preeclampsia is associated with widespread apoptosis of placental cytotrophoblasts within the uterine wall. Am J Pathol (1999) 155:293–301. doi: 10.1016/S0002-9440(10)65123-1 10393861 PMC1866652

[89] He G Xu W Chen Y Liu X Xi M. Abnormal apoptosis of trophoblastic cells is related to the up-regulation of CYP11A gene in placenta of preeclampsia patients. PloS One (2013) 8:e59609. doi: 10.1371/JOURNAL.PONE.0059609 23555723 PMC3612086

[90] Mor G Abrahams VM. Potential role of macrophages as immunoregulators of pregnancy. Reprod Biol Endocrinol (2003) 1:1–8. doi: 10.1186/1477-7827-1-119/FIGURES/4 PMC305335 14651752

[91] Joerink M Rindsjö E van Riel B Alm J Papadogiannakis N. Placental macrophage (Hofbauer cell) polarization is independent of maternal allergen-sensitization and presence of chorioamnionitis. Placenta (2011) 32:380–5. doi: 10.1016/J.PLACENTA.2011.02.003 21419483

[92] Satoh T Takeuchi O Vandenbon A Yasuda K Tanaka Y Kumagai Y. The Jmjd3-Irf4 axis regulates M2 macrophage polarization and host responses against helminth infection. Nat Immunol (2010) 11:936–44. doi: 10.1038/ni.1920 20729857

[93] Yeon MK Romero R Seo YO Chong JK Kilburn BA Armant DR. Toll-like receptor 4: A potential link between “danger signals,” the innate immune system, and preeclampsia? Am J Obstet Gynecol (2005) 193:921.e1–8. doi: 10.1016/J.AJOG.2005.07.076 16157088

[94] al Mamun A Chauhan A Qi S Ngwa C Xu Y Sharmeen R. Microglial IRF5-IRF4 regulatory axis regulates neuroinflammation after cerebral ischemia and impacts stroke outcomes. Proc Natl Acad Sci USA (2020) 117:1742–52. doi: 10.1073/PNAS.1914742117/-/DCSUPPLEMENTAL PMC6983422 31892541

[95] el Chartouni C Schwarzfischer L Rehli M. Interleukin-4 induced interferon regulatory factor (Irf) 4 participates in the regulation of alternative macrophage priming. Immunobiology (2010) 215:821–5. doi: 10.1016/J.IMBIO.2010.05.031 20580461

[96] Yang SW Cho EH Choi SY Lee YK Park JH Kim MK. DC-SIGN expression in hofbauer cells may play an important role in immune tolerance in fetal chorionic villi during the development of preeclampsia. J Reprod Immunol (2017) 124:30–7. doi: 10.1016/J.JRI.2017.09.012 29049918

[97] Deng B Wehling-Henricks M Villalta SA Wang Y Tidball JG. IL-10 triggers changes in macrophage phenotype that promote muscle growth and regeneration. J Immunol (2012) 189:3669–80. doi: 10.4049/JIMMUNOL.1103180 PMC3448810 22933625

[98] Sulahian TH Högger P Wahner AE Wardwell K Goulding NJ Sorg C. Human monocytes express cd163, which is upregulated by il-10 and identical TO p155. Cytokine (2000) 12:1312–21. doi: 10.1006/CYTO.2000.0720 10975989

[99] Gray MJ Poljakovic M Kepka-Lenhart D Morris SM. Induction of arginase I transcription by IL-4 requires a composite DNA response element for STAT6 and C/EBPβ. Gene (2005) 353:98–106. doi: 10.1016/J.GENE.2005.04.004 15922518

[100] Wu WK Georgiadis A Copland DA Liyanage S Luhmann UFO Robbie SJ. IL-4 regulates specific arg-1+ macrophage sFlt-1–mediated inhibition of angiogenesis. Am J Pathol (2015) 185:2324–35. doi: 10.1016/J.AJPATH.2015.04.013 26079814

[101] Lang R Patel D Morris JJ Rutschman RL Murray PJ. Shaping gene expression in activated and resting primary macrophages by IL-10. J Immunol (2002) 169:2253–63. doi: 10.4049/JIMMUNOL.169.5.2253 12193690

[102] Schottelius AJG Mayo MW Balfour Sartor R Baldwin AS. Interleukin-10 signaling blocks inhibitor of κB kinase activity and nuclear factor κB DNA binding *. J Biol Chem (1999) 274:31868–74. doi: 10.1074/JBC.274.45.31868 10542212

[103] Dalmas E Toubal A Alzaid F Blazek K Eames HL Lebozec K. Irf5 deficiency in macrophages promotes beneficial adipose tissue expansion and insulin sensitivity during obesity. Nat Med (2015) 21:610–8. doi: 10.1038/nm.3829 25939064

[104] Mosser DM Hamidzadeh K Goncalves R. Macrophages and the maintenance of homeostasis. Cell Mol Immunol (2020) 18:579–87. doi: 10.1038/s41423-020-00541-3 PMC7491045 32934339

[105] A-Gonzalez N Bensinger SJ Hong C Beceiro S Bradley MN Zelcer N. Apoptotic cells promote their own clearance and immune tolerance through activation of the nuclear receptor LXR. Immunity (2009) 31:245–58. doi: 10.1016/J.IMMUNI.2009.06.018 PMC2791787 19646905

[106] Kourtzelis I Hajishengallis G Chavakis T. Phagocytosis of apoptotic cells in resolution of inflammation. Front Immunol (2020) 11:553/BIBTEX. doi: 10.3389/FIMMU.2020.00553/BIBTEX 32296442 PMC7137555

[107] Gong D Shi W Yi S Chen H Groffen J Heisterkamp N. TGFβ signaling plays a critical role in promoting alternative macrophage activation. BMC Immunol (2012) 13:1–10. doi: 10.1186/1471-2172-13-31 22703233 PMC3406960

[108] Oishi Y Manabe I. Macrophages in inflammation, repair and regeneration. Int Immunol (2018) 30:511–28. doi: 10.1093/INTIMM/DXY054 30165385

[109] Hanania R Sun HS Xu K Pustylnik S Jeganathan S Harrison RE. Classically activated macrophages use stable microtubules for matrix metalloproteinase-9 (MMP-9) secretion. J Biol Chem (2012) 287:8468–83. doi: 10.1074/JBC.M111.290676/ATTACHMENT/13099993-EBF4-49E0-A71C-77C3089A9E06/MMC1.PDF PMC3318683 22270361

[110] Xu X Zheng L Yuan Q Zhen G Crane JL Zhou X. Transforming growth factor-β in stem cells and tissue homeostasis. Bone Res (2018) 6:1–31. doi: 10.1038/s41413-017-0005-4 29423331 PMC5802812

[111] Kobayashi T Kim HJ Liu X Sugiura H Kohyama T Fang Q. Matrix metalloproteinase-9 activates TGF-β and stimulates fibroblast contraction of collagen gels. Am J Physiol Lung Cell Mol Physiol (2014) 306:L1006. doi: 10.1152/AJPLUNG.00015.2014 24705725 PMC4042193

[112] Zajac E Schweighofer B Kupriyanova TA Juncker-Jensen A Minder P Quigley JP. Angiogenic capacity of M1- and M2-polarized macrophages is determined by the levels of TIMP-1 complexed with their secreted proMMP-9. Blood (2013) 1122(25):4054–67. doi: 10.1182/BLOOD-2013-05-501494 PMC3862278 24174628

