# Supplementary material for: Placental macrophages present distinct polarization pattern and effector functions depending on clinical onset of preeclampsia
Source: Front Immunol. 2023 Jan 12;13:1095879. doi: 10.3389/fimmu.2022.1095879 (PMC9878680; doi:10.3389/fimmu.2022.1095879)
Supplement: Supplementary file 1 [file DataSheet_1.pdf]

Supplementary Material

Supplementary methods

Isolation of human arterial endothelial cells (fpEC)

Primary fpECs were isolated from chorionic arterial blood vessels (CTR n=5, EO-PE n=3) as described by Lang et al (51). Isolated fpECs were cultured at 12% oxygen and 37 °C on 1% gelatin-coated flasks (Sigma Aldrich) using Endothelial Cell Growth Medium MV (Promocell) supplemented with 5% FCS (Promocell), hydrocortisone (Promocell), ECGS (Promocell), and 1% gentamicin (Thermo Fischer Scientific).

Proliferation assay

BrDU

To measure the proliferation of EO-PE and CTR fpEC, BrDU colorimetric cell proliferation ELISA kit (Roche) was used according to the manufacturer´s recommendation. fpEC were seeded overnight at a cell density of $2x10^6$ cells/mL in Endothelial Cell Growth Medium MV (Promocell). The medium was then replaced with a pool of conditioned medium (CM) obtained from 10 different CTR and 5 EO-PE HBC isolations. CM was diluted 1:1 with full fpEC medium. Incubated macrophage medium without cells diluted with endothelial medium served as a control. After 24 hours of treatment with CM, 10 µL of BrDU reagent was added and incubated for 3 hours. After fixation, denaturation and incubation with the anti-BRdU- POD working solution, cells were intensively washed and in final step incubated with the substrate solution for 25 minutes. Afterwards, absorbance was measured at 492 nm using the SPECTROstar Nano plate reader (BMG Lab Technologies).

MTS

This assay (Cell titer 96 Aquaeous one solution cell, Promega) was applied according to the manufacturer's instructions. EO-PE and CTR fpEC were seeded in a gelatin-coated 96-well plate at a cell density of $1x10^6$ cells/mL in Endothelial Cell Growth Medium MV containing supplements (Promocell) and cultured overnight. The medium was then replaced with a pool of CM obtained from 10 different CTR and 5 EO-PE HBC isolations. The CM was diluted 1:1 with the complete fpEC medium. Incubated macrophage medium without HBCs, diluted with fpEC medium served as a control. After 24 hours of treatment, 20 µL of MTS reagent was added to the cells and incubated for 4 hours. Absorbance was measured at 492 nm using the SPECTROstar Nano plate reader (BMG Labtech).

Supplementary Tables

Table S1: List of antibodies and their concentrations used for the FACS analysis

Antibody	Distributor / Catalog number	Concentration
APC anti-human CD163 Antibody	BioLegend / 333609	15 µl / 100 µl
PE Mouse Anti-Human CD68	BD Pharmingen™ / 556078	10 µl / 100 µl
V450 Mouse Anti-Human CD11b	BD Pharmingen™ / 560481	5 µl / 100 µl
PE Mouse Anti-Human CD11c	BD Pharmingen™ / 560999	5 µl / 100 µl
FITC Mouse Anti-Human CD206	BD Pharmingen™ / 551135	5 µl / 100 µl
PerCP-Cy™5.5 Mouse Anti-Human CD209	BD Pharmingen™ / 558263	10 µl / 100 µl
V450 Mouse Anti-Human CD80	BD Pharmingen™ / 560442	10 µl / 100 µl
V450 Mouse Anti-Human CD86	BD Pharmingen™ / 560357	5 µl / 100 µl
FITC Anti-TIL/TLR1 antibody	Abcam / ab59702	5 µl / 100 µl
Human TLR4 PE-conjugated Antibody	R&D / FAB6248P	5 µl / 100 µl
Pacific Blue™ anti-human HLA-DR Antibody	BioLegend / 307633	5 µl / 100 µl
APC anti-human Folate Receptor β (FR-β) Antibody	BioLegend / 391705	2 µl / 100 µl
FITC Anti-TLR2 antibody	abcam / ab59711	3 µl / 100 µl
PE Mouse Anti- IRF4	BD Pharmingen™ / 566646	5 µl / 100 µl

IRF5 Monoclonal Antibody (ALYSCLN), eFluor 660	Invitrogen / 50-9698- 41	5 µl / 100 µl
FITC Mouse Anti-Human CD40	BD Pharmingen™ / 556624	5 µl / 100 µl
7-AAD	BD Pharmingen™ / 559925	5 µl / 100 µl
FITC Mouse IgG1 K Isotype Control	BD Pharmingen™/ 555748	5 µl / 100 µl
PerCP-Cy™5.5 Mouse IgG1 κ Isotype Control	BD Pharmingen™/ 550795	10 µl / 100 µl
Pacific Blue™ Mouse IgG1, κ Isotype Ctrl Antibody	BioLegend / 400151	5 µl / 100 µl
V450 Mouse IgG1/ κ Isotype Control	BD Pharmingen™ / 560373	5 µl / 100 µl
APC Mouse IgG1 K Isotype Control	BD Pharmingen™/ 555751	10 µl / 100 µl
PE Mouse IgG1 K Isotype Control	BD Pharmingen™/ 555749	5 µl / 100 µl

Table S2: List of primer assays used for the RT-qPCR analysis

Target	Assay	Supplier
RPL30	Hs_RPL30_1_SG QuantiTect Primer Assay (QT00056651)	Quiagen
HPRT1	Hs_HPRT1_1_SG QuantiTect Primer Assay (QT00059066)	Quiagen
MMP9	Hs_MMP9_1_SG QuantiTect Primer Assay (QT00040040)	Quiagen

MMP12	Hs_MMP12_1_SG QuantiTect Primer Assay (QT01004472)	Quiagen
TIMP1	Hs_TIMP1_1_SG QuantiTect Primer Assay (QT00084168)	Quiagen
TIMP2	Hs_TIMP2_1_SG QuantiTect Primer Assay (QT00017759)	Quiagen
CCL4	Hs_CCL4_1_SG QuantiTect Primer Assay (QT01008070)	Quiagen
IL6	Hs_IL6_1_SG QuantiTect Primer Assay (QT00083720)	Quiagen
CXCL8	Hs_CXCL8_1_SG QuantiTect Primer Assay (QT00000322)	Quiagen
ICAM	Hs_ICAM1_1_SG QuantiTect Primer Assay (QT00074900)	Quiagen
VCAM	Hs_VCAM1_1_SG QuantiTect Primer Assay (QT00018347)	Quiagen
TGFB1	Hs_TGFB1_1_SG QuantiTect Primer Assay (QT00000728)	Quiagen
IL10	Hs_IL10_1_SG QuantiTect Primer Assay (QT00041685)	Quiagen
18S	Forward (5’-3’) CTACCACATCCAAGGAAGCA<br>Reverse (5’-3’) TTTTTCGTCACTACCTCCCCG	Sigma- Aldrich
NFKB1	KiCqStart™ Primer: H_NFKB1_1	Sigma- Aldrich
LEP	KiCqStart™ Primer: H_LEP_1	Sigma- Aldrich
HIF1A	KiCqStart™ Primer: H_HIF1A_1	Sigma- Aldrich
SOD	KiCqStart™ Primer: H_SOD1_1	Sigma- Aldrich

CAT	KiCqStart™ Primer: H_CAT_1	Sigma- Aldrich
ARG1	KiCqStart™ Primer: H_ARG1_1	Sigma- Aldrich
IL1A	KiCqStart™ Primer: H_IL1A_1	Sigma- Aldrich
IL1B	KiCqStart™ Primer: H_IL1B_1	Sigma- Aldrich
TNF	KiCqStart™ Primer: H_TNF_1	Sigma- Aldrich
FLT1	KiCqStart™ Primer: H_FLT1_1	Sigma- Aldrich
VEGFA	KiCqStart™ Primer: H_VEGFA_1	Sigma- Aldrich
KDR	KiCqStart™ Primer: H_KDR_1	Sigma- Aldrich
CDH2	KiCqStart™ Primer: H_CDH2_1	Sigma- Aldrich
CDH5	KiCqStart™ Primer: H_CDH5_1	Sigma- Aldrich
NOS2	KiCqStart™ Primer: H_NOS2_1	Sigma- Aldrich

Supplementary Figures

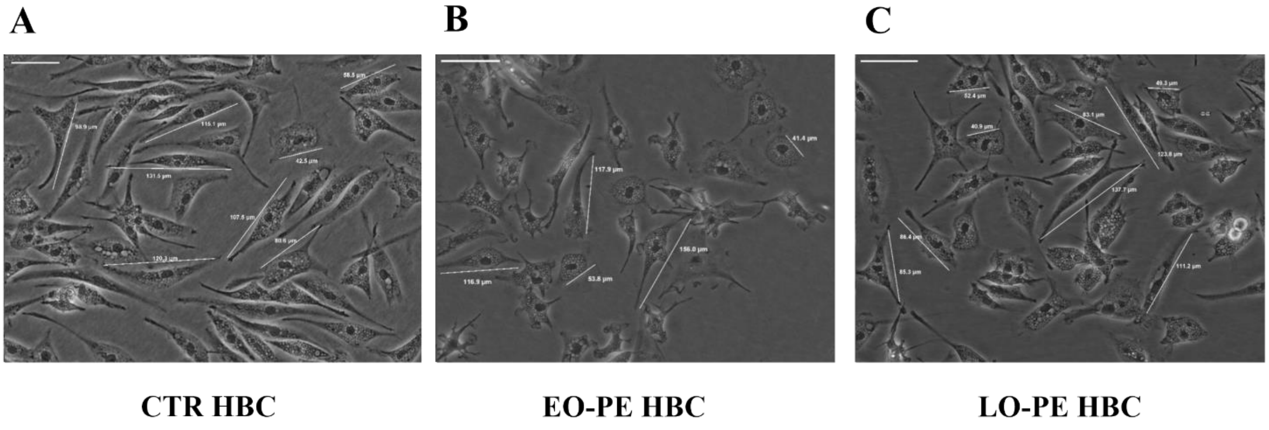

Figure S1: Cell morphology and characterization of CTR (A), early onset (EO-) (B) and late onset (LO-) PE (C) HBCs isolated from term placentae. Representative images of HBCs taken on the 5th day post isolation are shown. Scale bar represents 50µm, cell size was measured using CellSens measuring tool.

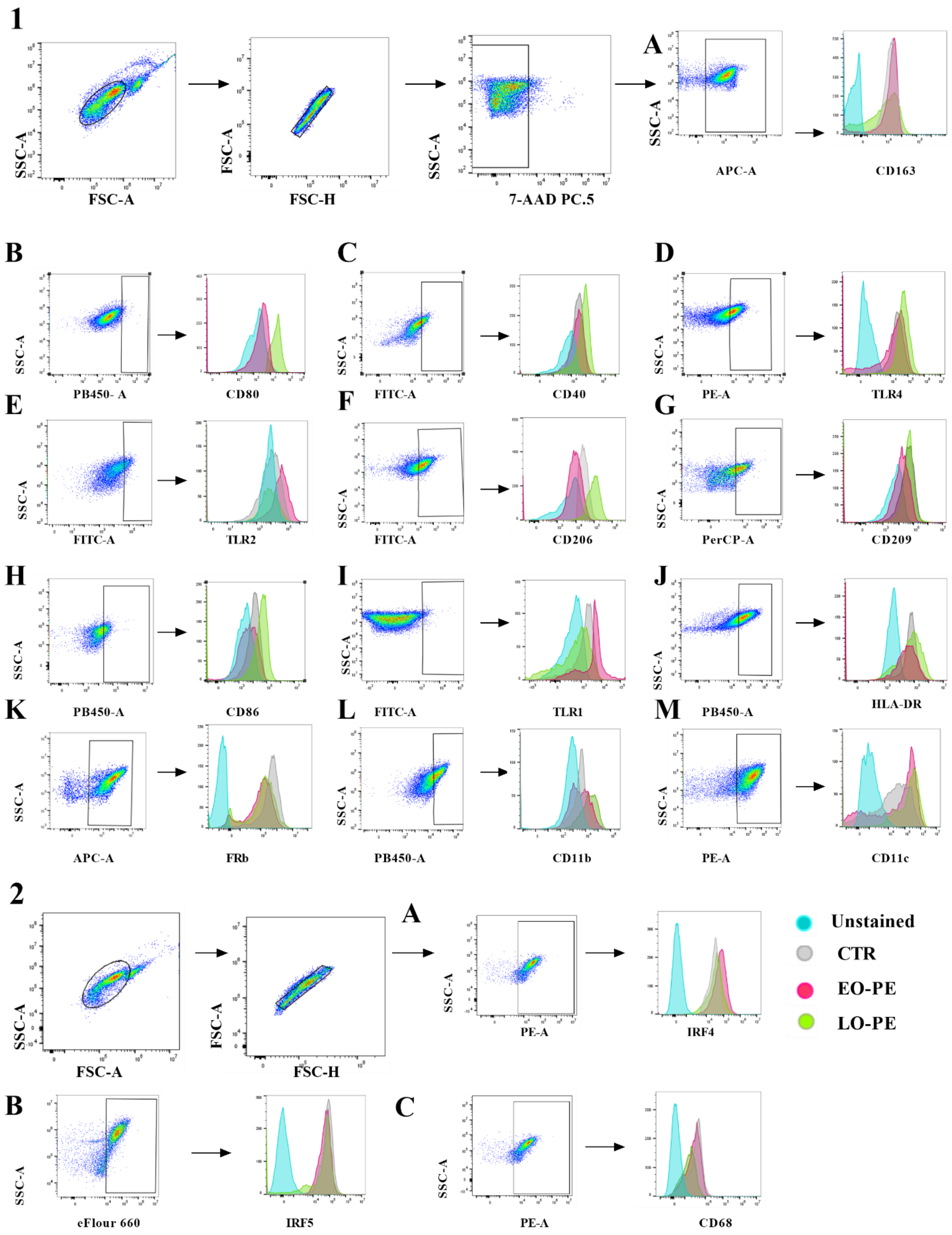
1
SSC-A
FSC-A
FSC-A
FSC-H
SSC-A
7-AAD PC.5
A
SSC-A
APC-A
CD163
B
SSC-A
PB450- A
CD80
C
SSC-A
FITC-A
CD40
D
SSC-A
PE-A
TLR4
E
SSC-A
FITC-A
TLR2
F
SSC-A
FITC-A
CD206
G
SSC-A
PerCP-A
CD209
H
SSC-A
PB450-A
CD86
I
SSC-A
FITC-A
TLR1
J
SSC-A
PB450-A
HLA-DR
K
SSC-A
APC-A
FRb
L
SSC-A
PB450-A
CD11b
M
SSC-A
PE-A
CD11c
2
SSC-A
FSC-A
FSC-A
FSC-H
A
SSC-A
PE-A
IRF4
Unstained
CTR
EO-PE
LO-PE
B
SSC-A
eFlour 660
IRF5
C
SSC-A
PE-A
CD68

Figure S2: Representative gating strategy to identify the expression of specific surface (1) and intracellular (2) polarization markers. (1A) CD163, (1B) CD80, (1C) CD40, (1D) TLR4, (1E) TLR2, (1F) CD206, (1G) CD209, (1H) CD86, (1I) TLR1, (1J) HLA-DR, (1K) folate receptor-β (FRb), (1L) CD11b, (1M) CD11c, respectively. (2) Staining against intracellular markers: (2A) IRF4, IRF5 (2B), and (2C) CD68, respectively. Blue histogram peaks represent unstained samples, grey represents CTR HBCs, red represents EO-PE HBCs and green represents LO-PE HBCs. Histograms of one representative experiment are shown, in total a number of isolations of CTR (n=14), EO (n=6) and LO (n=5) were used.

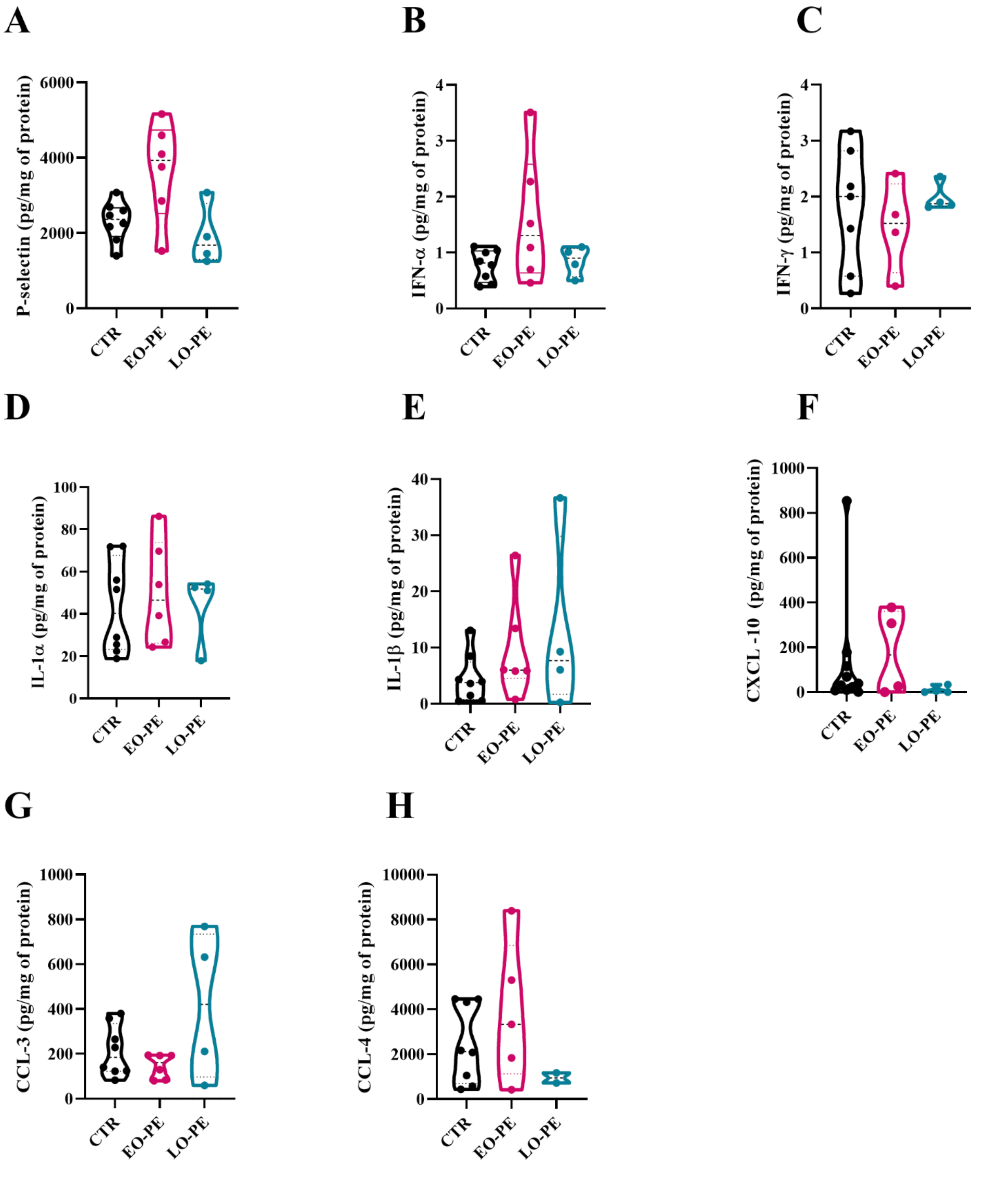

Figure S3: Secretion of pro- and anti-inflammatory cytokines CTR (n=8), EO- (n=6) and LO-PE (n=4) HBCs. Multiplex-ELISA-on-beads assay for followed pro-inflammatory and anti-inflammatory cytokines (A) P-selectin, (B) IFN-α, (C) IFN- γ, (D) IL-1β, (E) IL-17a, (F) CXCL-10, (G) CCL-3 and (H) CCL-4, respectively. Multiplex was performed in duplicates. Secretion of respective cytokines

was normalized to the total protein content measured in the cell culture supernatants. Statistical significance was assessed using ANCOVA with adjustment for gestational age followed by Sidak´s post-hoc test. *p ≤ 0.05.

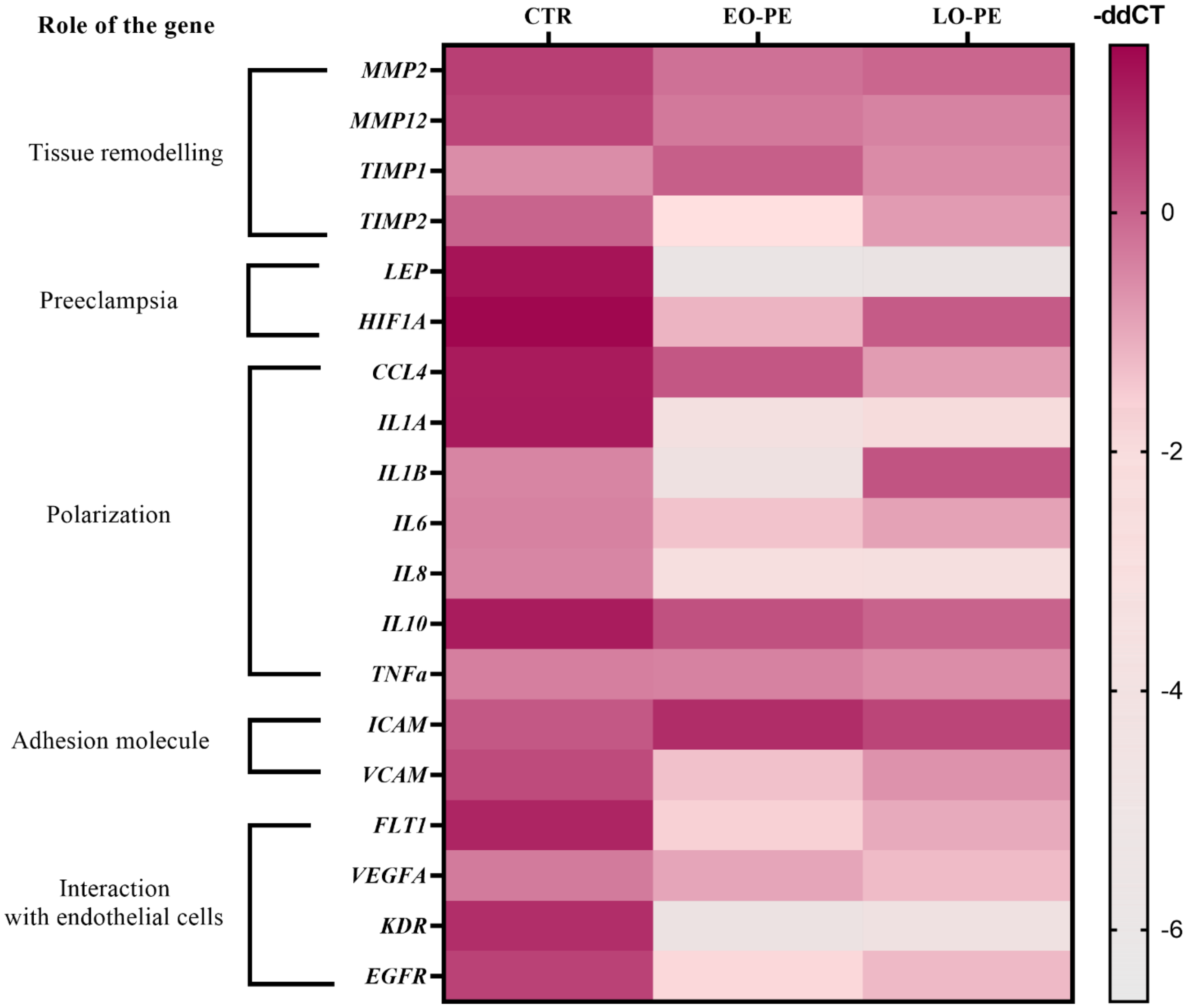

Figure S4: Preeclampsia alters inflammatory related gene expression in HBCs. Total RNA of cultivated HBCs was harvested on the fifth day post isolation and analysed by RT-qPCR. Genes associated with PE, inflammation and regulation of polarization are shown on the heat map. In total 12 CTR, 5 EO-PE and 5 LO-PE HBCs isolations in three technical replicates were used. Expression of target genes was normalized to the following housekeeping genes (*18S*, *RPL30* and *HPRT1*) using $2^{\Delta\Delta Ct}$ method. Statistical significance was tested using ANCOVA with adjustment for gestational age followed by Sidak's post-hoc test. *p ≤ 0.05.

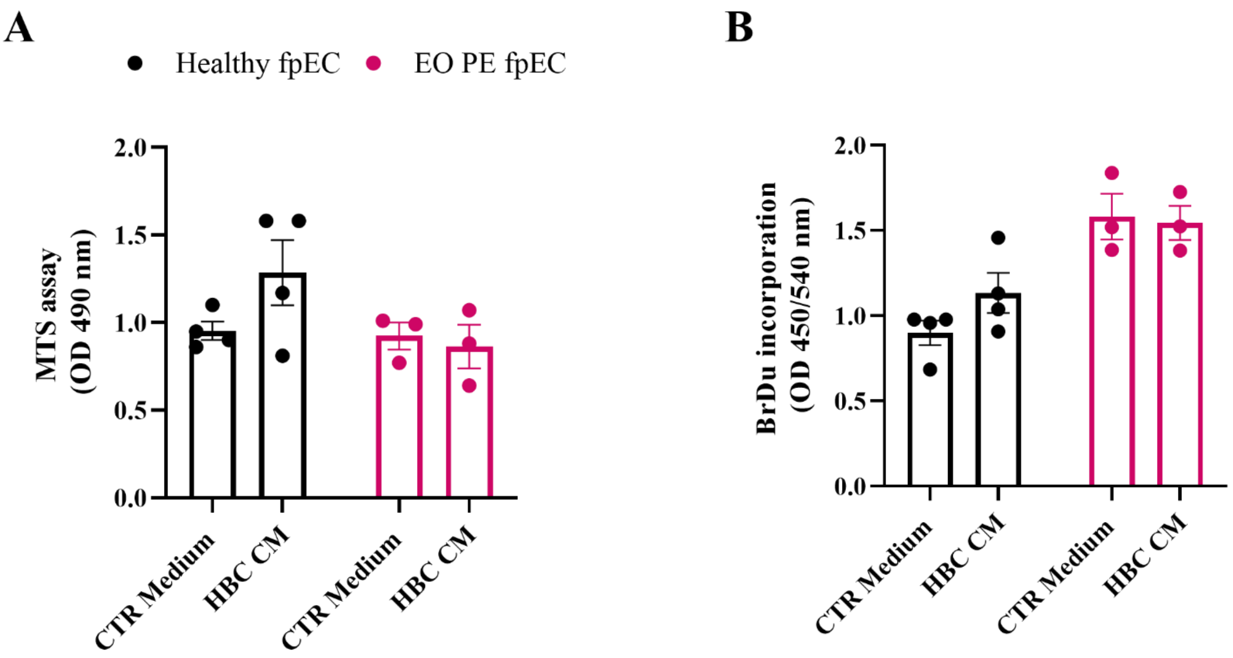

Figure S5: Effect of CTR and EO-PE conditioned medium (CM) of HBCs on the proliferation of CTR and PE fpECA. CM from 5 different HBC isolations was used for proliferation assays with CTR (n=4) and PE (n=3) fpECA. As a CTR medium cultivated Macrophage medium without cells was used. Healthy fpEC were treated with CM collected from healthy HBCs and PE fpEC with CM collected from EO-PE HBCs, respectively. CM of CTR HBCs tend to have an effect on proliferation of fpEC measured with MTS assay (A) and BrDU assay (B). Data are presented as the mean of the technical triplicates of the number of individual biological replicates of fpECA. To test statistical significance Two-way ANOVA with Sidak's post-hoc test was used. Data are shown as mean ± S.E.M. p-value ≤ 0.05 was considered statistically significant.

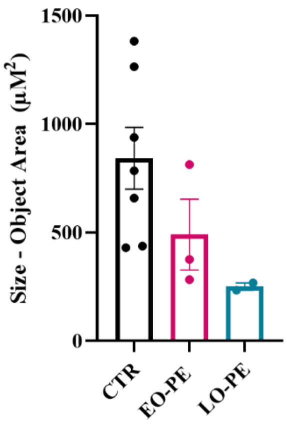

Figure S6: Measurement of morphology (size-object area, µM^2) using high content screening (HCS). Measurement of the size of the HBCs was performed using NisViewer software. All data in are

presented as mean ± S.E.M, ANCOVA with adjustment for gestational age with Sidak's post-hoc test was used for to test statistical significance. *$p \leq 0.05$.
